# Acute myeloid leukemia mitochondria hydrolyze ATP to support oxidative metabolism and resist chemotherapy

**DOI:** 10.1126/sciadv.adu5511

**Published:** 2025-04-09

**Authors:** James T. Hagen, McLane M. Montgomery, Raphael T. Aruleba, Brett R. Chrest, Polina Krassovskaia, Thomas D. Green, Emely A. Pacheco, Miki Kassai, Tonya N. Zeczycki, Cameron A. Schmidt, Debajit Bhowmick, Su-Fern Tan, David J. Feith, Charles E. Chalfant, Thomas P. Loughran, Darla Liles, Mark D. Minden, Aaron D. Schimmer, Md Salman Shakil, Matthew J. McBride, Myles C. Cabot, Joseph M. McClung, Kelsey H. Fisher-Wellman

**Affiliations:** ^1^ Department of Physiology, Brody School of Medicine, East Carolina University, Greenville, NC, USA.; ^2^ East Carolina Diabetes and Obesity Institute, East Carolina University, Greenville, NC, USA.; ^3^ Department of Cancer Biology, Atrium Health Wake Forest Baptist Comprehensive Cancer, Wake Forest University School of Medicine, Winston-Salem, NC, USA.; ^4^ Department of Biochemistry and Molecular Biology, Brody School of Medicine, East Carolina University, Greenville, NC, USA.; ^5^ Department of Biology, East Carolina University, Greenville, NC, USA.; ^6^ Brody School of Medicine at East Carolina University, Flow Cytometry Core, Greenville, NC, USA.; ^7^ Department of Medicine, Hematology/Oncology, University of Virginia School of Medicine, Charlottesville, VA, USA.; ^8^ University of Virginia Cancer Center, Charlottesville, VA, USA.; ^9^ Department of Cell Biology, University of Virginia, Charlottesville, VA, USA.; ^10^ Research Service, Richmond Veterans Administration Medical Center, Richmond, VA, USA.; ^11^ Department of Internal Medicine, Brody School of Medicine, East Carolina University, Greenville, NC, USA.; ^12^ Princess Margaret Cancer Centre, University Health Network, Toronto, Canada.; ^13^ Department of Chemical Biology, Ernest Mario School of Pharmacy, Rutgers University, Piscataway, NJ, USA.; ^14^ Rutgers Cancer Institute, Rutgers University, New Brunswick, NJ, USA.; ^15^ Section of Molecular Medicine, Department of Internal Medicine, Wake Forest University School of Medicine, Winston-Salem, NC, USA.

## Abstract

OxPhos inhibitors have struggled to show a clinical benefit because of their inability to distinguish healthy from cancerous mitochondria. Herein, we describe an actionable bioenergetic mechanism unique to acute myeloid leukemia (AML) mitochondria. Unlike healthy cells that couple respiration to ATP synthesis, AML mitochondria support inner-membrane polarization by consuming ATP. Matrix ATP consumption allows cells to survive bioenergetic stress. Thus, we hypothesized AML cells may resist chemotherapy-induced cell death by reversing the ATP synthase reaction. In support, BCL-2 inhibition with venetoclax abolished OxPhos flux without affecting mitochondrial polarization. In surviving AML cells, sustained mitochondrial polarization depended on matrix ATP consumption. Mitochondrial ATP consumption was further enhanced in AML cells made refractory to venetoclax, consequential to down-regulations in the endogenous F_1_-ATPase inhibitor ATP5IF1. Knockdown of ATP5IF1 conferred venetoclax resistance, while ATP5IF1 overexpression impaired F_1_-ATPase activity and heightened sensitivity to venetoclax. These data identify matrix ATP consumption as a cancer cell–intrinsic bioenergetic vulnerability actionable in the context of BCL-2 targeted chemotherapy.

## Teaser

Unlike healthy blood cells, AML cells reverse the ATP synthase reaction to support oxidative metabolism and resist chemotherapy.

## INTRODUCTION

Similar to a growing list of human solid organ malignancies, acute myeloid leukemia (AML) is particularly reliant on oxidative metabolism (*1*–*7*). Pioneering studies over the past several years have repeatedly demonstrated potent antileukemic effects upon targeting AML’s mitochondrial reliance (*8*–*16*). Although mitochondrial targeted therapies are entering clinical trials, most hopeful “mito-therapeutics” suffer from a limited therapeutic index based on their inability to discriminate cancerous from noncancerous mitochondria (*16*–*20*). The lack of cancer-specific mitochondrial targets poses a barrier to progress, as safe and effective mitochondrial-targeted therapies will likely require cancer cell selectivity. Fortunately, new evidence is emerging that all mitochondria are not alike. Mitochondria are unique in composition and function within each cell type, including AML cells (*16*–*19*, *21*–*24*). This raises the exciting possibility that identifying the unique bioenergetic signature(s) of AML cells may hold the key to designing mitochondrial-targeted therapies that preferentially eradicate the malignant cells.

Compared to normal blood cells, AML cells present with intrinsic deficiencies in oxidative phosphorylation (OxPhos) (*23*). Despite AML cells having a larger mitochondrial network, the fraction of this network capable of coupling respiration to the synthesis of ATP is less than 50% (*23*). This fraction has been found to fall below 30% in chemoresistant AML (*25*). Thus, although oxidative adenosine 5′-triphosphate (ATP) synthesis is essential for most all eukaryotic life, there is emerging evidence linking impaired OxPhos function to the biology of AML, particularly chemoresistant AML. With respect to chemoresistant disease, despite intrinsic OxPhos limitations, mitochondrial membrane potential (ΔΨ_m_) is either sustained or hyperpolarized in drug-resistant AML cells (*2*, *26*). Mitochondrial hyperpolarization typically tracks with high OxPhos. Thus, the low OxPhos yet hyperpolarized phenotype of drug-resistant AML mitochondria is unexpected and, as such, diagnostic of cell-intrinsic mitochondrial remodeling (*27*, *28*).

Mitochondrial polarization is canonically generated via the proton pumping respiratory complexes (i.e., complexes I/III/IV). In respiratory incompetent cells, polarization can also be sustained via cytosol to matrix ATP transport and/or ATP hydrolysis by the reversible ATP synthase reaction (*29*–*33*). Thus, in the absence of a sufficient proton-motive force, ATP synthase reverts to an F_1_–adenosine triphosphatase (ATPase), converting the free energy available in the cell’s phosphate potential to a polarized mitochondrial inner membrane (*29*, *30*, *32*, *34*). Maintenance of ΔΨ_m_ via matrix F_1_-ATPase activity has been demonstrated to be critical for cell survival in the context of respiratory complex lesions (*29*, *34*). Mitochondrial depolarization is a key trigger for apoptosis (*35*, *36*); therefore, mitochondrial ATP consumption may also limit the cytotoxicity of AML cells in response to OxPhos damaging chemotherapy.

Designed to directly initiate the intrinsic pathway of apoptosis at the mitochondria (*37*), the targeted therapy venetoclax (i.e., a BCL-2 inhibitor and a mediator of BAX/BAK-dependent apoptosis) was granted regular Food and Drug Administration approval for the treatment of AML in 2020 (*38*). Despite improving patient outcomes, clinical efficacy of venetoclax remains stifled by the development of chemoresistant disease (*39*–*41*). Prior research has established that venetoclax exposure inhibits AML mitochondrial respiration (*42*) by impinging on components of the electron transport system (ETS) (*43*). Although respiration is inhibited by venetoclax, ΔΨ_m_ is less affected (*8*, *26*). How AML cells sustain ΔΨ_m_ in the presence of venetoclax and whether sustained ΔΨ_m_ drives venetoclax resistance remain unclear. To investigate this, we leveraged an in-house mitochondrial phenotyping platform to systematically define the interplay between venetoclax exposure, mitochondrial bioenergetics, and AML cell survival.

## RESULTS

### Intrinsic lesions in respiratory complex IV limit OxPhos flux in AML cells

**Fig. 1.**
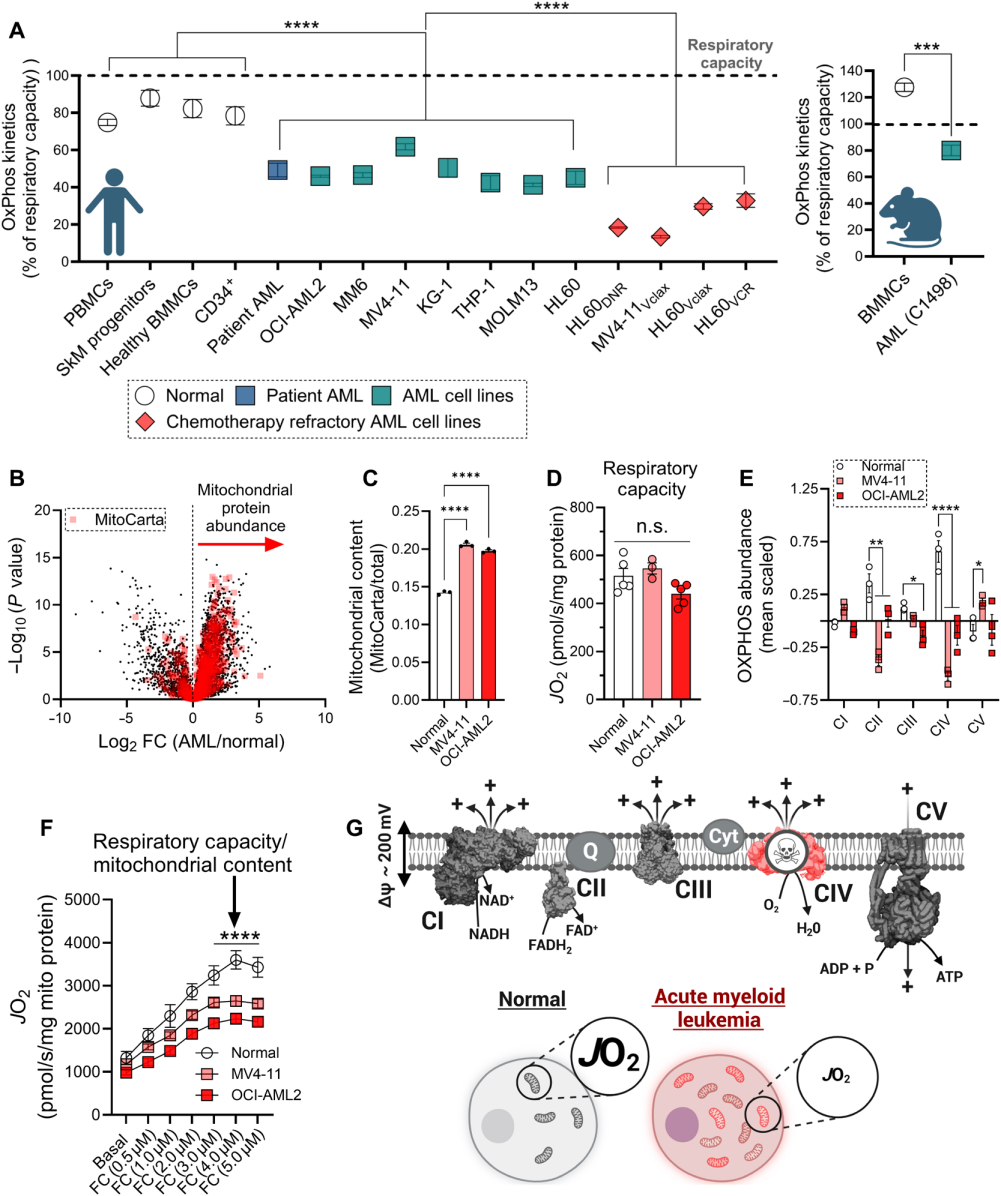
Intrinsic lesions in respiratory complex IV limit OxPhos flux in AML cells. All experiments were performed using whole cells and digitonin-permeabilized cells. (**A**) Comparison of fractional OxPhos of normal blood cells, patient AML, AML cell lines, chemotherapy refractory AML cell lines, and mouse AML cells calculated as the ratio of *J*H^+^_OXPHOS_ to *J*H^+^_Total_ (*n* = 3 to 30 replicates) and represented as a percentage of total respiratory capacity. Skeletal muscle (SkM) progenitors refer to myoblasts isolated from human skeletal muscle biopsy. (**B**) Volcano plot comparing mitochondrial and non-mitochondrial proteome of AML cells and healthy bone marrow mononuclear cells (BMMCs). Log_2_FC, log_2_ fold change. (**C**) Comparison of mitochondrial content in AML cells and healthy BMMCs (*n* = 3 to 30 replicates). (**D**) Comparison of whole-cell respiratory capacity in healthy BMMCs, MV4-11 cells, and OCI-AML2 cells normalized to milligrams of protein (*n* = 3 to 5 replicates). n.s., not significant. (**E**) Comparison of the OxPhos proteome between healthy BMMCs and AML cells (*n* = 3 replicates). (**F**) Comparison of whole-cell respiratory capacity in healthy BMMCs, MV4-11 cells, and OCI-AML2 cells normalized to milligrams of mitochondrial protein (*n* = 3 to 5 replicates). (**G**) Schematic depicting complex IV lesions and reduced respiration of individual AML mitochondria. Data are presented as means ± SEM and analyzed by two-way ANOVA [(E) and (F)] and one-way ANOVA [(A), (C), and (D)]. *P < 0.05; **P < 0.01; ***P < 0.001; ****P < 0.0001. NAD^+^, nicotinamide adenine dinucleotide (oxidized form); NADH (reduced form); FAD^+^, flavin adenine dinucleotide (oxidized form); FADH_2_ (reduced form). (A) and (G) created using BioRender.com.

Relative to normal myeloid progenitor cells, AML blasts are characterized by high mitochondrial content (*6*, *23*). Despite having more mitochondria per cell, we previously demonstrated that less than half of the AML mitochondrial network appears capable of coupling respiration to the synthesis of ATP, a phenomenon indicative of OxPhos limitations (*23*). To assess whether this OxPhos insufficiency is consistent across various AML models, we compared OxPhos capacity to total respiratory capacity in a panel of AML cell lines (both human and mouse) and patient samples. Our analysis revealed that, across all AML models, only 50% or less of the total respiratory flux (“respiratory capacity”) is used for OxPhos (Fig. 1A) (fig. S1, A to H). Notably, in AML cell lines that were made refractory to chemotherapy, the OxPhos insufficiency was even more pronounced (Fig. 1A). Such findings indicate that intrinsic limitations in OxPhos flux are a consistent bioenergetic feature of AML cells, with potential implications for the biology of treatment resistance.

To explore the underlying mechanisms of OxPhos limitations in AML, we compared OxPhos complex expression and OxPhos respiratory flux between AML cell lines (e.g., MV4-11 and OCI-AML2) and healthy myeloid progenitors isolated from bone marrow aspirates. As reported previously (*6*, *23*), mitochondrial protein abundance was higher in AML blasts, corresponding to a ~40% increase in mitochondrial content (Fig. 1, B and C). High mitochondrial content would be expected to lead to a corresponding increase in cellular respiratory capacity. However, protein normalized respiratory capacity was not elevated in AML blasts (Fig. 1D), indicating discordance between mitochondrial network size and respiratory kinetics, a feature diagnostic of respiratory complex lesions. To investigate the intrinsic composition of the respiratory system in AML, the expression of each respiratory complex and ATP synthase (i.e., the OxPhos components) were normalized to mitochondrial content. In doing so, mean scaled abundance of several respiratory complexes was observed to be lower in AML cells (Fig. 1E) (fig. S1, I to M). Complex specific decreases were most pronounced for cytochrome oxidase (i.e., complex IV), the terminal O_2_-consuming enzyme in the ETS (Fig. 1E). Consistent with lower ETS expression, cellular respiratory capacity per unit of mitochondrial protein was lower in AML cells (Fig. 1F). These data agree with prior reports in patient AML samples showing lower specific activity of the terminal proton-pumping respiratory complexes (*6*). Moreover, AML-specific lesions in complexes III and IV were recently shown to limit OxPhos flux in mouse AML cells (*21*). Together, these data implicate reduced OxPhos function as a conserved bioenergetic feature of AML cells driven by intrinsic lesions in the proton pumping respiratory complexes. AML cells appear to circumvent these ETS lesions to sustain baseline respiratory flux by expanding mitochondrial content (Fig. 1G).

### AML mitochondria consume cellular ATP to support mitochondrial polarization

**Fig. 2.**
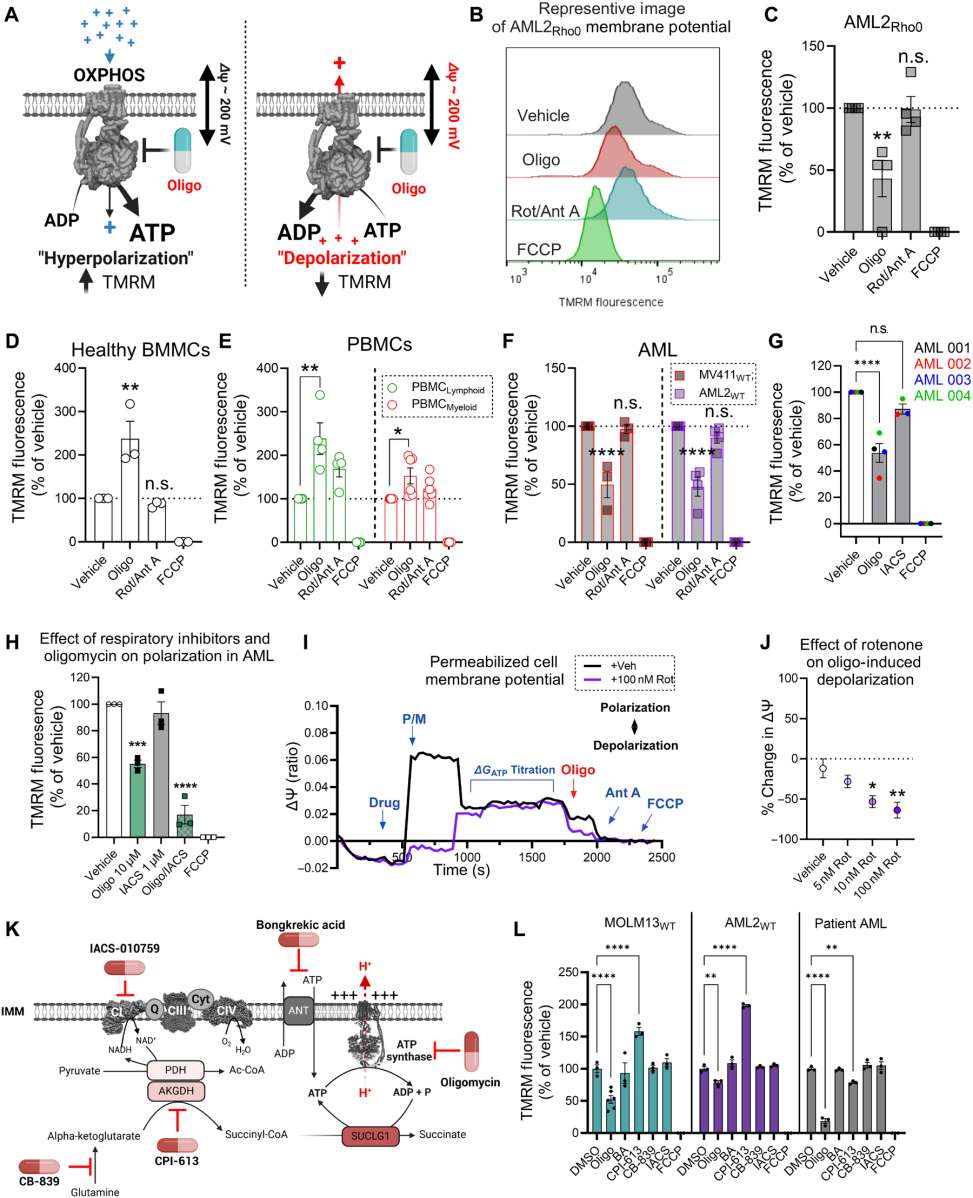
Mitochondria in AML cells consume cellular ATP to sustain mitochondrial polarization. All experiments were performed using whole intact cells. (**A**) Schematic depicting strategy to determine functional OxPhos in ΔΨ_m_ assays. (**B**) Representative image from flow cytometric analysis of intact cell ΔΨ_m_ in AML2_Rho0_ cells. (**C**) Flow cytometric analysis of intact cell ΔΨ_m_ in AML2_Rho0_ cells (*n* = 4 replicates). (**D**) Flow cytometric analysis of intact cell ΔΨ_m_ in healthy BMMCs (*n* = 3 replicates). (**E**) Flow cytometric analysis of intact cell ΔΨ_m_ in myeloid and lymphoid populations sorted from PBMCs (*n* = 4 to 6 replicates). (**F**) Flow cytometric analysis of intact cell ΔΨ_m_ in AML cell lines (*n* = 3 to 4 replicates). (**G**). Flow cytometric analysis of intact cell ΔΨ_m_ in patient AML (*n* = 4 patients). (**H**) Flow cytometric analysis of intact cell ΔΨ_m_ in pooled MV411_WT_, HL60_WT_, and AML2_WT_ cells (*n* = 1 replicate per cell type). (**I**) Representative trace of permeabilized cell ΔΨ_m_ assay in AML2_WT_ cells. (**J**) Comparison of oligomycin-induced depolarization in AML2_WT_ cells in the presence of increasing doses of rotenone (*n* = 4 replicates). (**K**) Schematic of mitochondrial inhibitors and their targets. (**L**) Flow cytometric analysis of intact cell ΔΨ_m_ in MOLM13_WT_, AML2_WT_, and patient AML cells exposed to the following inhibitors: oligomycin (10 μM), BA (50 μM), CPI-613 (200 μM), CB-839 (5 μM), IACS (0.5 μM), and FCCP (10 μM). Data are presented as means ± SEM and analyzed by two-way ANOVA (F), one-way ANOVA [(C), (D), (G), (H), (J), and (L)], or unpaired *t* test (E). *P < 0.05; **P < 0.01; ***P < 0.001; ****P < 0.0001. (A) and (K) created using BioRender.com.

Despite deficiencies in OxPhos, AML cells have more mitochondria than normal cells and those mitochondria generate a voltage potential (*2*, *23*, *44*). The ΔΨ_m_ can become polarized in two different ways: (i) via the proton-pumping respiratory complexes, complexes I/III/IV; or (ii) matrix ATP uptake via the adenine nucleotide translocase (ANT) and/or hydrolysis of ATP by the F_1_-ATPase (*45*). Because AML cells present with intrinsic respiratory complex lesions that would be expected to weaken the voltage generated by the ETS, we hypothesized that polarization may be supported, at least in part, via F_1_-ATPase activity. To test this, we used flow cytometry to assess mitochondrial polarization [using tetramethylrhodamine methyl ester (TMRM) fluorescence] in individual AML cells in the absence and presence of the ATP synthase inhibitor oligomycin. Operating in non-quench mode, an increase in TMRM fluorescence with oligomycin was taken to indicate functional OxPhos, whereas a decrease in TMRM fluorescence with oligomycin was assumed to indicate that ΔΨ_m_ was being generated, in part, via F_1_-ATPase activity (Fig. 2A). To validate our assay, ΔΨ_m_ was assessed in respiration-incompetent ρ^0^ OCI-AML2 (“AML2_Rho0_”) cells that present with a lack of mitochondrial respiration and a hyper-reduced reduced form of nicotinamide adenine dinucleotide (NADH) pool (fig. S2, A and B). Despite being depleted of mitochondrial DNA, ρ^0^ cells sustain ΔΨ_m_ by consuming ATP (*29*, *46*). In agreement, inhibition of ATP synthase with oligomycin triggered depolarization in AML2 _Rho0_ cells (Fig. 2, B and C). In mononuclear cells isolated from healthy bone marrow, as well as peripheral blood mononuclear cells (PBMCs) from healthy volunteers, the addition of oligomycin increased TMRM fluorescence, suggesting functional OxPhos (Fig. 2, D and E). ΔΨ_m_ was sustained in the presence of rotenone (i.e., a complex I inhibitor) and antimycin A (i.e., an inhibitor of complex III), indicating that normal blood cells can sustain ΔΨ_m_ via noncanonical mechanisms such as matrix ATP consumption when respiration is compromised (Fig. 2, D and E).

Having established the validity of our membrane potential assay, we next assessed ΔΨ_m_ in AML cells. Across seven AML cell lines with different founding mutations, as well as patient AML samples, the addition of oligomycin lowered TMRM fluorescence (Fig. 2, F and G, and fig. S2, C to G). The magnitude of depolarization in AML cells upon ATP synthase inhibition was comparable to that seen in respiration-deficient AML2 _Rho0_ cells (Fig. 2C). Using both oligomycin to block ATP synthase and IACS-010759 (IACS) to block the ETS, near-complete depolarization was achieved (Fig. 2H and fig. S2, C to G), suggesting that both the ETS and ATP consumption cooperate to sustain ΔΨ_m_. To control for effects driven by constituents in Iscove’s Modified Dulbecco’s Medium (IMDM) culture medium, ΔΨ_m_ in AML2_WT_ cells was assessed in a culture medium formulated to mimic the physiological profile of human plasma [i.e., human plasma-like medium (HPLM)]. Oligomycin also triggered depolarization in AML2_WT_ cells assayed in HPLM (fig. S2H). To further validate, fluorescent microscopy was used to measure changes in TMRM fluorescence (operating in non-quench mode) of MV411_WT_ cells in response to either oligomycin, rotenone, and antimycin A or those in combination. Single-agent oligomycin depolarized ΔΨ_m_ in MV411_WT_ cells (fig. S2, I and J). Rotenone and antimycin A also resulted in depolarization; however, when oligomycin, rotenone, and antimycin A were used in combination, the magnitude of depolarization was similar to that of alamethicin, a channel forming peptide antibiotic that eliminates ΔΨ_m_ (fig. S2, I and J).

Our data suggest that, in AML mitochondria, ATP consumption is secondary to intrinsic deficiencies in the ETS. These ETS deficiencies constrain the ability of the proton-pumping respiratory complexes to maintain a ΔΨ_m_ sufficient to oppose proton pumping by ATP synthase. If this is correct, then ATP hydrolysis should become progressively more important for maintaining ΔΨ_m_ in response to progressive ETS loss of function. To model this, we energized permeabilized AML2_WT_ cells with pyruvate and malate, ensuring NADH oxidation at complex I was the sole source of electrons. This setup allowed us to gradually add a complex I inhibitor, rotenone, to evaluate how reduced ETS functionality affected mitochondrial polarization through ATP hydrolysis. We measured ΔΨ_m_ using TMRM fluorescence in quench mode, across various ATP/adenosine 5′-diphosphate (ADP) ratios, with Δ*G*_ATP_ values ranging from −54.2 to −61.5 kJ/mol. After titrating Δ*G*_ATP_, oligomycin was added to inhibit F1-ATPase activity, and changes in TMRM fluorescence were quantified. A drop in ΔΨ_m_ after oligomycin addition reflects the extent to which ATP hydrolysis supports inner membrane polarization. The results showed that rotenone dose-dependently inhibited complex I–driven polarization, which correlated with a greater depolarization when oligomycin was added (Fig. 2, I and J, and fig. S2K). During the assay, increased exposure to Δ*G*_ATP_ reduced ETS flux, either through direct inhibition by ATP or indirectly by the ΔΨ_m_ generated from ATP synthase, which counteracts proton pumping by the ETS. Together, these findings support a model where damage to the ETS, which limits the proton-pumping activity of respiratory complexes, increases the reliance on F1-ATPase activity to maintain polarization in AML cells.

Regarding the source of ATP being consumed by AML cells, we considered both glycolysis (in the cytosol) and substrate-level phosphorylation (in the matrix). Substrate-level phosphorylation in the matrix is catalyzed by succinyl–coenzyme A (CoA) synthetase, which couples the conversion of succinyl-CoA to succinate with the phosphorylation of ADP to form ATP. Succinyl-CoA is generated during the oxidation of branched-chain keto acids and alpha-ketoglutarate, the latter of which is produced via the glutaminolysis pathway. To block substrate-level phosphorylation, we treated AML cells with the glutaminase inhibitor CB-839 or CPI-613. CPI-613 inhibits lipoate-dependent dehydrogenase complexes in the matrix, including alpha-ketoglutarate dehydrogenase (*47*, *48*). To inhibit glycolytic ATP production, we used 2-deoxyglucose (2-DG) or the cell-permeable inhibitor bongkrekic acid (BA), which targets ANT. All experiments also included single-agent treatments with IACS to assess the contribution of the ETS to ΔΨ_m_, as well as oligomycin (Fig. 2K). As observed in other AML cell lines, IACS did not affect ΔΨ_m_, while oligomycin partially collapsed ΔΨ_m_ in all cells (Fig. 2L). If substrate-level phosphorylation was supplying ATP for ΔΨ_m_, then treatment with CB-839 or CPI-613 would be expected to depolarize ΔΨ_m_. However, in two AML cell lines and patient-derived AML cells, CB-839 did not affect ΔΨ_m_ (Fig. 2L). At doses that inhibited alpha-ketoglutarate-supported respiration (fig. S2L), CPI-613 led to ΔΨ_m_ hyperpolarization in the AML cell lines (Fig. 2L). In patient AML cells, single-agent CPI-613 partially depolarized ΔΨ_m_ (Fig. 2L), suggesting substrate-level phosphorylation as a potential ATP source. Across all cell models, blocking glycolytic ATP production with 2-DG or blocking cytosol to matrix ATP exchange with BA had no impact on ΔΨ_m_ (fig. S2M and Fig. 2L). These results suggest that ΔΨ_m_ in AML cells is maintained independent of glycolysis. However, we cannot exclude contributions from glycolysis, as the ATP required for F1-ATPase activity likely originates from multiple sources, and disruption of one pathway may be compensated by increased flux through others. For example, in respiration-deficient AML2_Rho0_ cells, blocking ANT with BA partially depolarized the ΔΨ_m_ (fig. S2N), consistent with a need for glycolytic ATP to support inner mitochondrial membrane polarization during conditions of extreme respiratory limitations. In addition, we did not use a specific inhibitor of succinyl-CoA synthetase; thus, we cannot rule out a contribution from succinyl-CoA derived from branched-chain keto acid oxidation. Overall, our findings suggest that, unlike normal blood cells with functional OxPhos, mitochondria in AML cells consume ATP to maintain ΔΨ_m_ polarization, with the necessary ATP likely originating from multiple sources.

### Matrix F_1_-ATPase activity supports oxidative metabolism in AML cells

**Fig. 3.**
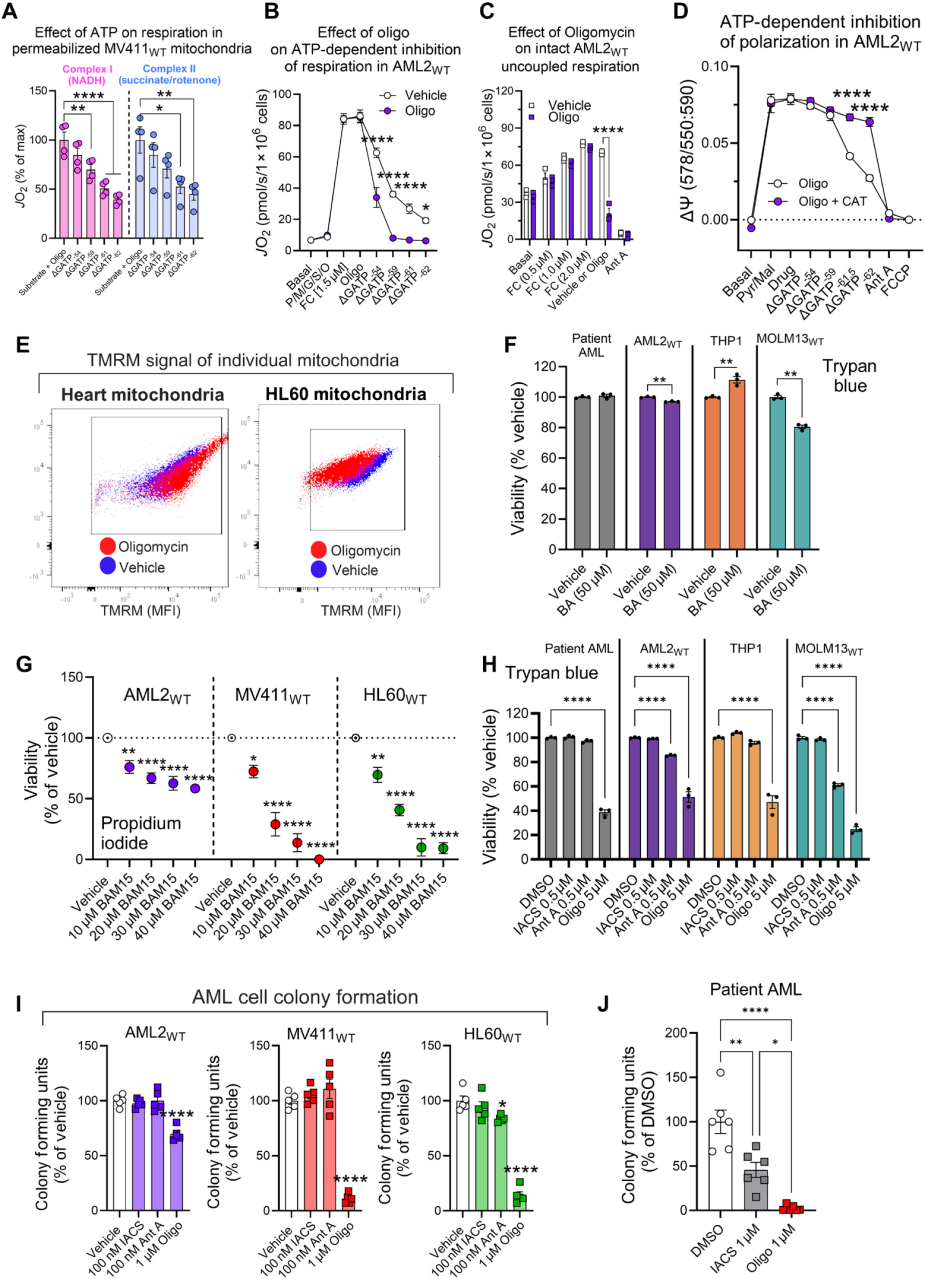
F_1_-ATPase activity supports both oxidative metabolism and survival in AML cells. All experiments were performed using whole intact cells, digitonin-permeabilized cells, or alamethicin-permeabilized mitochondria. (**A**) Effect of Δ*G*_ATP_ on maximal NADH (complex I) or succinate/rotenone (complex II) supported respiration in alamethicin-permeabilized MV411_WT_ mitochondria (*n* = 4 replicates). (**B**) Effect of oligomycin on inhibition of uncoupled respiration by Δ*G*_ATP_ in permeabilized AML2_WT_ cells (*n* = 3 replicates). (**C**) Effect of oligomycin on uncoupled respiration in intact AML2_WT_ cells (*n* = 3 replicates). (**D**) Effect of Δ*G*_ATP_ on polarization by pyruvate/malate (Pyr/Mal) in the presence of oligomycin and in the presence or absence of carboxyatractyloside (CAT; 5 μM), in permeabilized AML2_WT_ cells (*n* = 3 replicates). (**E**) Change in TMRM fluorescence of individual mitochondria in mitochondrial populations isolated from mouse heart or HL60 cells before and after oligomycin addition during flow cytometric analysis of the ΔΨ_m_. MFI, mean fluorescence intensity. (**F**) Cell viability, assessed via trypan blue, in AML cell lines and patient AML cells exposed to vehicle or BA (50 μM) for 48 hours (*n* = 3 replicates). (**G**) Effect of increasing doses of BAM15 uncoupler on AML2_WT_, MV411_WT_, and HL60_WT_ cell viability. Viability was measured using PI (*n* = 4 to 5 replicates). (**H**) Effect of IACS, antimycin A, or oligomycin on AML cell viability. Viability was measured using trypan blue (*n* = 3 replicates). (**I**) Effect of IACS, antimycin A, or oligomycin on AML2_WT_, MV411_WT_, and HL60_WT_ colony formation. Colonies were manually counted using light microscopy (*n* = 5 replicates). (**J**) Colony-forming unit assay in patient AML cells exposed to DMSO, IACS, or oligomycin (*n* = 6). Colonies quantified using CellTiter-Glo. Data are presented as means ± SEM and analyzed by two-way ANOVA [(A) to (D), (G), and (H)], one-way ANOVA [(I) and (J)], or unpaired *t* test (F). *P < 0.05; **P < 0.01; ****P < 0.0001.

In AML cells, mitochondria appear to be able to both respire and simultaneously reverse the ATP synthase reaction. Upon inhibition of F1-ATPase activity with oligomycin, the ETS alone fails to sustain ΔΨ_m_. These findings are unexpected, as they suggest that inhibition of F1-ATPase in AML cells also prevents polarization driven by the ETS. We have previously reported that respiration in AML cells is inhibited by matrix ATP (*23*). Building on these results, we found that, when MV4-11 mitochondria were permeabilized with alamethicin, uncoupled respiration supported by complex I (NADH) and complex II (succinate) substrates was dose-dependently inhibited by Δ*G*_ATP_ (Fig. 3A, fig. S3, A and B). Because the presence of oligomycin ensures that ATP synthase is nonfunctional during the assay, these data suggest that matrix ATP directly inhibits respiration in AML cells. F1-ATPase consumes matrix ATP. Thus, we hypothesized that F1-ATPase activity is crucial for maintaining respiration in AML cells by clearing matrix ATP. To test, digitonin-permeabilized AML2_WT_ cells were energized with saturating carbon substrates [pyruvate, malate, glutamate, succinate, and octanoyl-carnitine (“P/M/G/S/O”)], and respiration was stimulated with carbonyl cyanide ptrifluoromethoxyphenylhydrazone (FCCP). Cells were then treated with either dimethyl sulfoxide (DMSO) or oligomycin, and Δ*G*_ATP_ was titrated to inhibit respiration. In the presence of oligomycin, AML2_WT_ cell respiration was more sensitive to inhibition by Δ*G*_ATP_, supporting a model in which F1-ATPase activity supports respiration by clearing matrix ATP (Fig. 3B). To confirm that F1-ATPase activity also supports respiration in living AML cells, we stimulated intact AML2_WT_ cells with FCCP to eliminate any ability of ATP synthase to synthesize ATP and then treated them with either oligomycin or DMSO. Oligomycin inhibited uncoupled respiration in intact AML2_WT_ cells (Fig. 3C), further suggesting that F1-ATPase activity clears matrix ATP to support respiration in living AML cells. Given that matrix ATP inhibits mitochondrial respiration in AML, we hypothesized that it might also affect ETS polarization. To test this, we assessed the impact of ATP on the mitochondrial membrane potential in permeabilized AML2_WT_ cells. All conditions included oligomycin to prevent ATP synthase from clearing any added ATP. Experimental conditions involved pyruvate/malate to drive respiration-dependent polarization, with either no carboxyatractyloside (“CAT”) or CAT to inhibit ANT-mediated ATP uptake into the matrix. We then titrated Δ*G*_ATP_ to assess its effects. Titration of Δ*G*_ATP_ depolarized the mitochondrial inner membrane in AML2_WT_ cells, and this effect was rescued when we blocked the ability of the added ATP to enter the matrix with CAT (Fig. 3D). These results suggest that matrix ATP inhibits ETS polarization, implying that ΔΨ_m_ in AML cells is likely insufficient to support ANT-mediated ATP transport from the matrix to the cytosol. Thus, matrix ATP clearance by the F1-ATPase appears essential to prevent ATP from disrupting both respiration and mitochondrial inner membrane polarization.

It is possible that individual AML cells harbor two distinct mitochondrial populations: one that respires and synthesizes ATP, and another that cannot respire but consumes ATP. To investigate this hypothesis, we developed an assay to measure the mitochondrial membrane potential using TMRM fluorescence in non-quench mode. This approach allowed us to assess the impact of oligomycin on mitochondrial membrane potential under experimental conditions that favor OxPhos flux. In these experiments, mitochondria isolated from HL60 cells were compared with those from a tissue known for its high OxPhos reliance, cardiac mitochondria isolated from mice. After isolation, mitochondria were energized with pyruvate/malate, exposed to Δ*G*_ATP_ of −61.5 kJ/mol, followed by the addition of oligomycin. In cardiac mitochondria, oligomycin induced an increase in polarization, consistent with functional OxPhos, while, in HL60 cells, it caused a decrease in polarization (fig. S3, C and D). When TMRM fluorescence of individual mitochondria was plotted before (“vehicle”) and after oligomycin treatment (“oligomycin”), the fluorescence shifts were homogeneous in both cardiac and HL60 populations (Fig. 3E). These data suggest that AML cell mitochondria are capable of both respiration and simultaneous ATP consumption. Such findings are consistent with the reversal potentials of ANT relative to ATP synthase which allow for a weakened ΔΨ_m_ generated by the ETS to allow ATP synthase to run in reverse without catalyzing ADP/ATP exchange via ANT (*30*). Consistent with minimal ANT flux under basal conditions, exposure to a cell-permeable inhibitor of ANT (BA) did not affect ΔΨ_m_ (Fig. 2L) and minimally affected AML cell viability (Fig. 3F). Ultimately, F1-ATPase activity in AML cells seems crucial for degrading matrix ATP, regardless of its origin, to prevent depolarization.

### Blocking matrix F_1_-ATPase activity is cytotoxic to AML cells

Mitochondrial depolarization has been shown to be an upstream driver of cell death via apoptosis (*35*). When AML cells were exposed to increasing doses of the mitochondrial protonophore BAM15, dose-dependent toxicity was observed (Fig. 3G), consistent with ΔΨ_m_ being necessary for AML cell survival. Given our data linking F_1_-ATPase activity to sustained mitochondrial polarization in AML cells, we hypothesized that F_1_-ATPase activity is necessary for AML survival. To test this, AML cell viability and colony formation were quantified in response to inhibitors that would either block respiration without affecting F1-ATPase activity (e.g., IACS and antimycin A) or block F1-ATPase activity directly (e.g., oligomycin). As expected, IACS and antimycin A eliminated basal respiration in AML cells (fig. S3E). However, despite not being able to respire, AML cells were largely resistant to overt losses in cell viability and colony formation in response to either drug (Fig. 3, H and I), presumably because the cells could sustain polarization via ATP consumption. Consistent with this, oligomycin resulted in greater losses of cell viability and colony-forming capacity, indicating F_1_-ATPase activity is necessary for AML cell survival (Fig. 3, H and I). Although AML cells can polarize via both the ETS and the F_1_-ATPase, our data suggest that oligomycin results in an inhibition of polarization originating from both the F_1_-ATPase and the ETS (i.e., by inhibition of the respiratory complexes by matrix ATP). In support, in patient AML cells, colony formation was only partially blunted by the ETS inhibitor IACS, while oligomycin near-eliminated colony formation (Fig. 3J). Collectively, these data demonstrate that mitochondrial respiration is dispensable for AML cell survival and highlight matrix F_1_-ATPase activity an actionable vulnerability of AML cells.

### Venetoclax exposure collapses mitochondrial respiration, but polarization is sustained via F_1_-ATPase activity

**Fig. 4.**
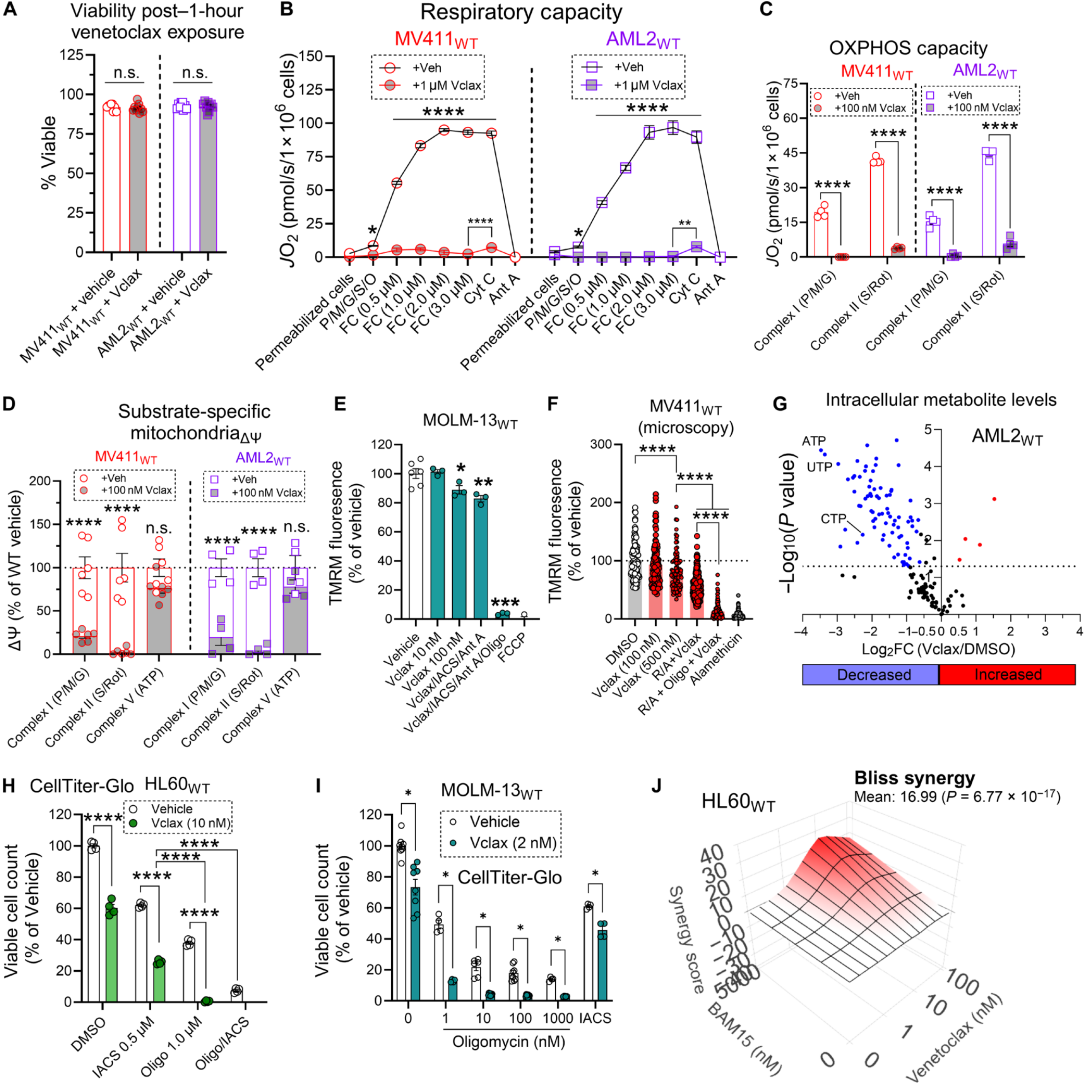
Venetoclax exposure collapses mitochondrial respiration, but polarization is sustained via F_1_-ATPase activity. (**A**) Effect of 1 hour exposure to venetoclax (0.1 and 1 μM doses combined) on AML cell viability (*n* = 9 replicates). (**B** and **C**) Effect of 1 hour exposure to 1 μM venetoclax on respiratory capacity (B) or OxPhos capacity (C) in permeabilized AML cells (*n* = 3 to 4 replicates). (**D**) Effect of 1 hour exposure to 100 nM venetoclax on polarization induced by complex I substrates (P/M/G), complex II substrates (S/R), or complex V substrates (ATP) in permeabilized AML cells (*n* = 4 to 6 replicates). (**E**) Flow cytometric analysis of intact MOLM-13_WT_ cell ΔΨ_m_ in response to single-agent venetoclax, and venetoclax in combination with IACS and antimycin A, and those in combination with oligomycin (*n* = 3 replicates). (**F**) Fluorescent microscopy analysis of intact MV411_WT_ cell ΔΨ_m_ in response to single-agent venetoclax, and venetoclax in combination with rotenone and antimycin A, and those in combination with oligomycin (*n* = 100 to 311 cells). (**G**) Volcano plot depicting changes in intracellular metabolites in OCI-AML2 cells exposed to DMSO or venetoclax (100 nM) for 1 hour (*n* = 3). CTP, cytidine 5′-triphosphate; UTP, uridine 5′-triphosphate. (**H**) Effect of single-agent venetoclax, venetoclax in combination with IACS and oligomycin, or IACS in combination with oligomycin on HL60_WT_ cell viability. Viability was measured using CellTiter-Glo (*n* = 4 replicates). (**I**) Effect of single-agent venetoclax or venetoclax in combination with either oligomycin or IACS on MOLM-13_WT_ cell viability. Viability was measured using CellTiter-Glo (*n* = 4 to 8 replicates). (**J**) Bliss Synergy analysis of cell viability in HL60_WT_ cells exposed venetoclax (0, 1, 10, and 100 nM) in the absence and presence of BAM15 (5 μM). Data are presented as means ± SEM and analyzed by two-way ANOVA [(C) to (E) and (H)], one-way ANOVA [(B) and (F)], or unpaired *t* test [(A) and (I)]. *P < 0.05; **P < 0.01; ***P < 0.001; ****P < 0.0001.

In the absence of compensatory mechanisms, ATP-mediated inhibition of respiration in the mitochondrial matrix likely promotes apoptosis in cells with mitochondrial damage by depolarizing the inner mitochondrial membrane. However, in AML cells, despite inherent defects in complex IV, cell survival is maintained through the degradation of matrix ATP by F1-ATPase. This process not only helps sustain mitochondrial polarization but also provides resistance to respiratory inhibition, enabling AML cells to bypass typical apoptotic pathways. Disruptions in AML cell respiration have been reported to occur in response to various chemotherapeutics including doxorubicin (*49*), cytarabine (*50*), and venetoclax (*42*). Venetoclax, a specific inhibitor of the antiapoptotic BCL-2 protein, has been shown to inhibit respiratory flux in AML cells by impinging on the proton-pumping respiratory complexes (*42*, *43*). In our hands, 1-hour exposure to venetoclax in treatment naïve AML cells (“MV411_WT_” and “AML2_WT_”) collapsed mitochondrial respiration (i.e., both respiratory capacity and OxPhos respiratory flux), without overtly reducing cell viability (Fig. 4, A to C) (fig. S4, A and B). Consistent with venetoclax mediating BAX/BAK-dependent outer mitochondrial membrane permeabilization (*37*), respiration of AML cells exposed to venetoclax was increased following the addition of cytochrome C, indicating that BCL-2 inhibition results in permeabilization of the outer mitochondrial membrane [Fig. 4B; compare “FC (3.0 μM)” to “Cyt C”]. Because venetoclax collapsed mitochondrial respiration in AML cells, we hypothesized that polarization may be sustained via F_1_-ATPase activity. To test this, we leveraged our permeabilized cell ΔΨ_m_ assay. In this assay, polarization induced by various substrates was measured following 1-hour exposure of treatment naïve AML cells to venetoclax. Venetoclax exposure resulted in an inhibition of polarization by complex I [pyruvate/malate/glutamate (“P/M/G”)] and complex II [succinate/rotenone (“S/Rot”)] substrates (Fig. 4D and fig. S4, C and D). However, polarization by F_1_-ATPase activity (“ATP”) was not overtly affected by venetoclax exposure (Fig. 4D and fig. S4, C and D), suggesting that F_1_-ATPase activity can sustain polarization across the inner membrane during BCL-2 inhibition.

Although venetoclax is an emerging frontline therapy for AML, the development of chemoresistant disease limits its clinical efficacy (*39*–*41*). In preclinical models, combining venetoclax with respiratory inhibitors (e.g., IACS) has shown promise in enhancing its effectiveness (*11*, *51*). However, our findings suggest that AML cells can maintain mitochondrial polarization via F1-ATPase activity during venetoclax exposure (Fig. 4D and fig. S4, C and D). This adaptation could undermine the therapeutic potential of venetoclax in combination with respiratory inhibitors. When AML cells (MOLM-13_WT_ and MV411_WT_) were exposed to venetoclax combined with respiratory inhibitors (such as IACS and antimycin A, or rotenone and antimycin A), they maintained ~90% of their basal polarization (Fig. 4, E and F, and fig. S4E). However, inhibition of polarization was observed when oligomycin was included in the treatment (Fig. 4, E and F, and fig. S4E). This suggests that AML cells exposed to venetoclax can sustain mitochondrial polarization through ATP hydrolysis. Notably, within just 1 hour of venetoclax exposure, ATP levels, among other nucleotide triphosphates, were markedly depleted in AML cells (Fig. 4G and fig. 4F), consistent with the activation of matrix ATP hydrolysis in the cellular response to venetoclax. On the basis of these observations, we hypothesized that combining oligomycin with venetoclax might enhance AML cell death. To test this, HL60_WT_ and MOLM-13_WT_ cells were treated with various combinations of venetoclax, oligomycin, and IACS. When oligomycin and IACS were used together, there was a marked enhancement of cell death, regardless of whether venetoclax was present (Fig. 4H), correlating with near-complete mitochondrial depolarization (Fig. 2H). The addition of IACS to venetoclax treatment increased cytotoxicity in both HL60_WT_ and MOLM-13_WT_ cells (Fig. 4, H and I), although some cells survived presumably by sustaining polarization via F1-ATPase activity (Fig. 4, E and F). In the presence of oligomycin, venetoclax cytotoxicity was enhanced, consistent with the ability of oligomycin to drive mitochondrial depolarization (Fig. 4, H and I). Further supporting the significance of mitochondrial polarization for AML cell survival, we found that dissipation of the mitochondrial membrane potential using BAM15 worked synergistically with venetoclax to induce AML cell death (Fig. 4J). Together, these findings suggest that mitochondrial ATP hydrolysis enables AML cells to initially evade venetoclax-induced death, thus laying the foundation for the development of venetoclax resistance.

### Chemoresistant AML mitochondria present with a phenotypic shift toward enhanced ATP consumption

**Fig. 5.**
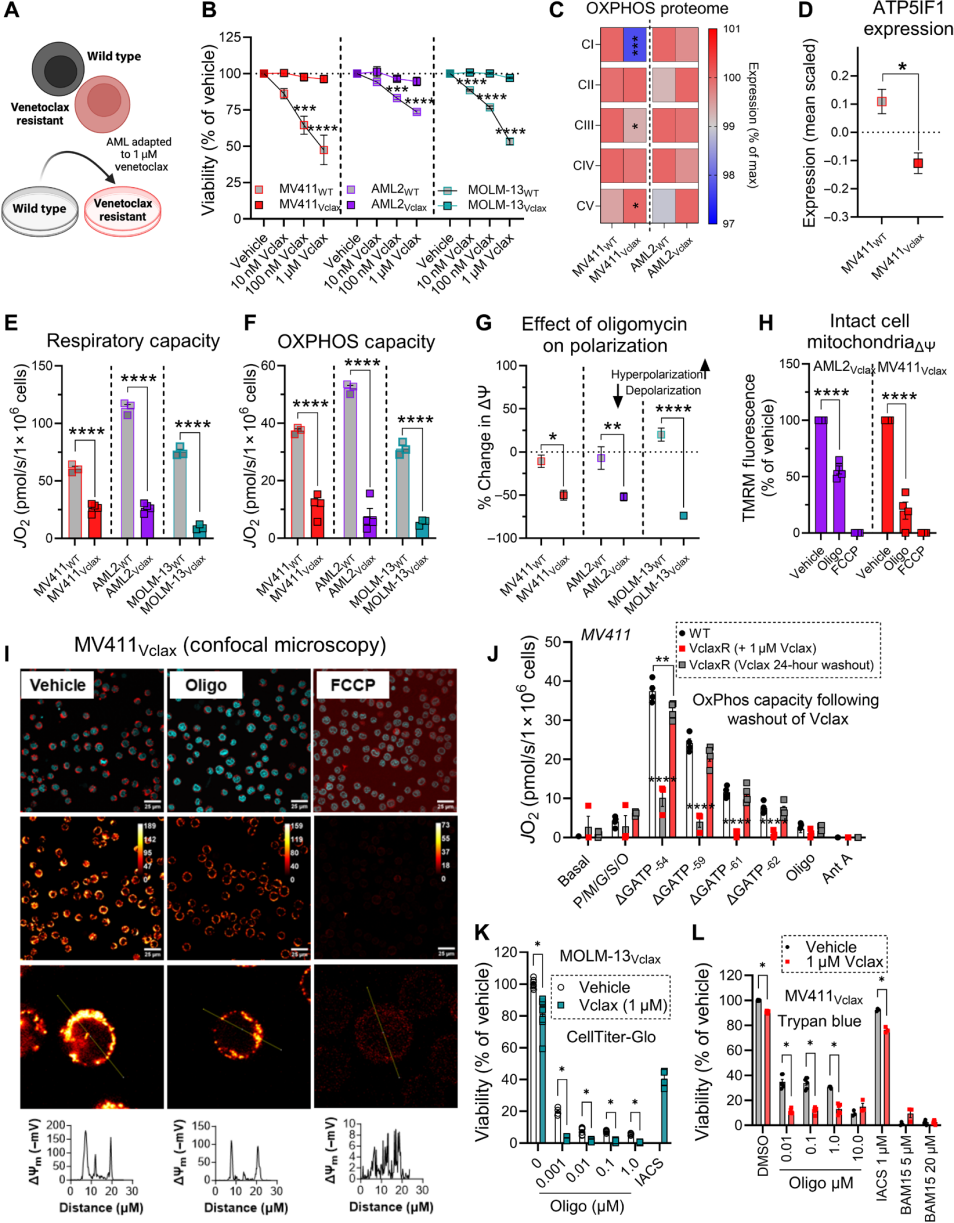
Chemoresistant AML mitochondria present with a phenotypic shift toward enhanced ATP consumption. (**A**) Schematic depicting generation of venetoclax-resistant AML cell lines. (**B**) Effect of venetoclax on treatment naïve AML and chemoresistant AML cell viability. Viability was measured using PI (*n* = 3 to 6 replicates). Created using BioRender.com. (**C**) Comparison of OxPhos proteome in permeabilized treatment naïve AML cells and venetoclax-resistant AML cells (*n* = 3 replicates). (**D**) Comparison of ATP5IF1 expression in mitochondria isolated from MV411_WT_ and MV411_Vclax_ cells (*n* = 3 replicates). (**E**) Comparison of respiratory capacity in permeabilized treatment naïve AML cells and venetoclax-resistant AML cells (*n* = 3 to 4 replicates). (**F**) Comparison of OxPhos capacity in permeabilized treatment naïve AML cells and venetoclax-resistant AML cells (*n* = 3 to 4 replicates). (**G**) Comparison of oligomycin-induced depolarization in permeabilized treatment naïve AML cells relative to permeabilized venetoclax-resistant AML cells (*n* = 3 to 6 replicates). (**H**) Flow cytometric analysis of intact cell ΔΨ_m_ in venetoclax-resistant AML cells (*n* = 4 replicates). (**I**) Confocal microscopy images and analysis of MV411_Vclax_ cells in the presence or absence of oligomycin. (**J**) Comparison of OxPhos kinetics in treatment naïve MV411_WT_ cells and MV411_Vclax_ cells grown in venetoclax or after venetoclax has been removed from culture medium for 24 hours (*n* = 4 replicates). (**K**) Effect of IACS and venetoclax or oligomycin in the presence or absence of venetoclax on MOLM-13_Vclax_ cell viability. Viability was measured using CellTiter-Glo (*n* = 4 to 8 replicates). (**L**) Effect of IACS and venetoclax, BAM15 and venetoclax, or oligomycin in the presence or absence of venetoclax on MV411_Vclax_ cell viability. Viability was measured using trypan blue (*n* = 3 replicates) Data are presented as means ± SEM and analyzed by two-way ANOVA [(B) and (J)], one-way ANOVA [(E) to (G)], or unpaired *t* test [(C), (D), (H), (K), and (L)]. *P < 0.05; **P < 0.01; ***P < 0.001; ****P < 0.0001.

Sustaining polarization via F_1_-ATPase activity appears to be an adaptation that opposes the intrinsic pathway of apoptosis, conferring survival to AML cells with reduced respiratory function regardless of the respiratory insult (e.g., by intrinsic complex IV lesions, ETS inhibitors, or venetoclax exposure). Given that venetoclax collapses mitochondrial respiration, we hypothesized that continuous exposure to the drug would positively select for chemoresistant AML clones that are more reliant on F_1_-ATPase activity to polarize the inner membrane. To test our hypothesis, we developed in vitro models of venetoclax resistance by culturing treatment naïve AML cells in venetoclax until they were refractory to 1 μM of the drug (MV411_Vclax_, AML2_Vclax_, and MOLM-13_Vclax_) (Fig. 5A). After multiple rounds of venetoclax selection in culture, a population of AML cells evolved that resisted apoptosis in response to venetoclax (Fig. 5B). Having established our in vitro models of venetoclax resistance, we then evaluated the impact of venetoclax resistance on mitochondrial proteome composition. The abundance of the proton-pumping respiratory complexes (complexes I/III/IV) were down-regulated in venetoclax-resistant AML cells, an adaptation expected to be deleterious to OxPhos function (Fig. 5C). Venetoclax-resistant cells up-regulated the abundance of the F_1_-ATPase/ATP synthase (complex V), suggesting an increase in demand for the total amount of F_1_-ATPase proton pumps (Fig. 5C). Analysis of isolated MV411_Vclax_ mitochondria revealed a down-regulation in the abundance of ATP5IF1, a known specific inhibitor (*52*–*54*) of ATP hydrolysis by ATP synthase (Fig. 5D).

To determine the impact of venetoclax resistance on AML mitochondrial bioenergetics, we compared respiratory capacity and OxPhos capacity of permeabilized treatment naïve AML cells and venetoclax-resistant AML cells. Venetoclax-resistant AML cells presented with a reduction in respiratory capacity and OxPhos capacity (Fig. 5, E and F, and fig. S5, A to F), consistent with down-regulations in complexes I/III/IV (Fig. 5C). Cytochrome C addition increased state 4 respiration in MOLM-13_Vclax_ cells, suggesting that venetoclax-resistant AML cells can resist apoptosis in response to venetoclax despite incurring damage to the outer mitochondrial membrane (fig. S5, C and F). We then compared ΔΨ_m_ of permeabilized venetoclax-resistant AML cells and treatment naïve AML cells. Polarization induced by pyruvate/malate was lower in venetoclax-resistant AML cells, and this tracked with a greater magnitude of depolarization by oligomycin (fig. S5, G to I, and Fig. 5G). To confirm that living venetoclax-resistant cells also sustain polarization via F_1_-ATPase activity, intact cell ΔΨ_m_ was assessed. Oligomycin triggered depolarization in living venetoclax-resistant AML cells, indicating that F_1_-ATPase activity sustains polarization (Fig. 5, H to I, and fig. S5J). Together, these data are consistent with our hypothesis that polarization via F_1_-ATPase activity is positively selected for in the setting of venetoclax selective pressure. Given that venetoclax collapses mitochondrial respiration in treatment naïve AML cells, we wanted to determine whether venetoclax also exerts bioenergetic effects in venetoclax-resistant AML cells. To test this, venetoclax was removed from MV411_Vclax_ culture medium for 24 hours. Following this, OxPhos capacity of MV411_Vclax_ cells was quantified and compared to that of MV411_WT_ cells. Although OxPhos capacity of MV411_WT_ cells remained higher than that of MV411_Vclax_ cells, the removal of venetoclax for 24 hours increased the OxPhos capacity of MV411_Vclax_ cells nearly threefold (Fig. 5J). Thus, even in chemoresistant cells, venetoclax retains its bioenergetic impact. We hypothesized that venetoclax-resistant AML cells can thus be resensitized to venetoclax by inhibiting F_1_-ATPase activity. To test this, the effect of oligomycin on both MOLM-13_Vclax_ and MV411_Vclax_ cell viability was measured in the presence or absence of venetoclax. Venetoclax in combination with oligomycin enhanced cell death of both cell lines (Fig. 5, K to L), consistent with restored sensitivity to venetoclax. Collectively, these data demonstrate that chemoresistant AML mitochondria present with a phenotypic shift toward enhanced ATP consumption, a potentially actionable bioenergetic mechanism critical for cells to evade venetoclax.

### Knockdown of ATP5IF1 to enhance matrix ATP consumption confers venetoclax resistance

**Fig. 6.**
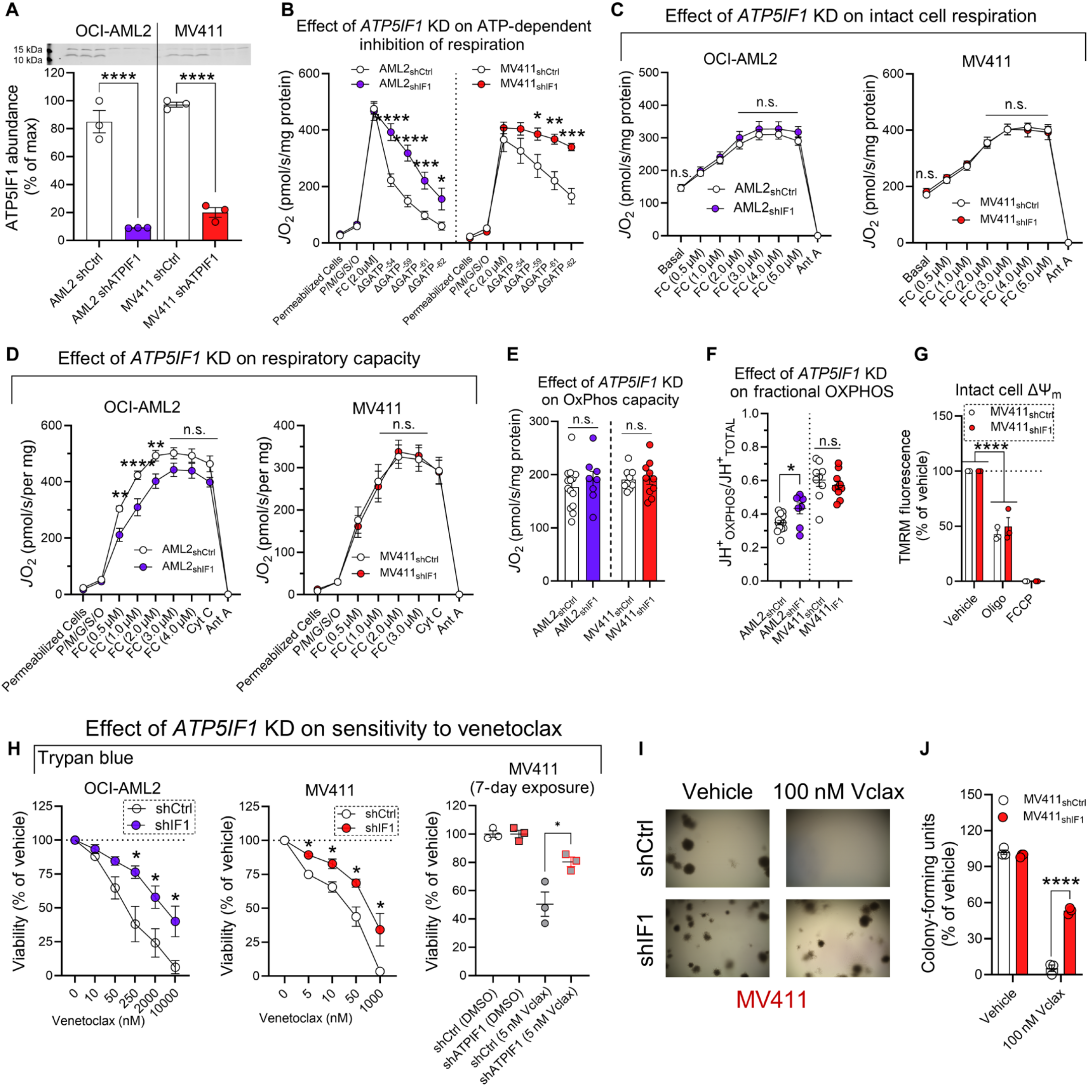
Knockdown of ATP5IF1 confers resistance to BCL-2 targeted therapy. (**A**) Expression of ATP5IF1 in isolated mitochondria (*n* = 3 replicates). (**B**) Impact of ATP5IF1 knockdown on the inhibition of respiration by Δ*G*_ATP_ in permeabilized scrambled short hairpin RNA (shRNA) control AML cells and permeabilized ATP5IF1 knockdown AML cells (*n* = 4 to 8 replicates). (**C**) Impact of ATP5IF1 knockdown on intact cell respiration of scrambled shRNA control AML cells and ATP5IF1 knockdown AML cells (*n* = 8 to 15 replicates). (**D**) Impact of ATP5IF1 knockdown (KD) on respiratory capacity in permeabilized scrambled shRNA control AML cells and permeabilized ATP5IF1 knockdown AML cells (*n* = 8 to 13 replicates). (**E**) Impact of ATP5IF1 knockdown on OxPhos capacity (*n* = 8 to 14 replicates). (**F**) Impact of ATP5IF1 knockdown on fractional OxPhos in permeabilized cells (*n* = 8 to 14 replicates). (**G**) Flow cytometric analysis of intact cell ΔΨ_m_ in MV411_shCtrl_ and MV411_shIF1_ cells (*n* = 3 replicates). (**H**) Effect of increasing doses of venetoclax on viability of scrambled shRNA control AML cells and ATP5IF1 knockdown AML cells after 48 hours, or the effect of venetoclax on MV411_shCtrl_ and MV411_shIF1_ viability after 7 days of exposure to venetoclax. Viability measured using trypan blue viable cell count (*n* = 4 replicates). (**I** and **J**) Effect of venetoclax on colony formation of MV411_shCtrl_ cells and MV411_shIF1_ cells. Colony formation was quantified using CellTiter-Glo (*n* = 3 replicates). Representative images of colony formation of MV411_shCtrl_ cells and MV411_shIF1_ cells in the presence or absence of venetoclax in (I). Data are presented as means ± SEM and analyzed by two-way ANOVA [(B) to (D) and (G)], one-way ANOVA [(A) and (E)], or unpaired *t* test [(F), (H), and (J)]. *P < 0.05; **P < 0.01; ***P < 0.001; ****P < 0.0001.

Continuous exposure to the mitochondrial damaging chemotherapeutic venetoclax positively selected for chemoresistant AML cells with the following characteristics: (i) up-regulated expression of the mitochondrial F_1_-ATPase and (ii) down-regulation of ATP5IF1. Given that ATP5IF1 specifically inhibits ATP hydrolysis by the F_1_-ATPase (*52*–*54*), we hypothesized that knockdown of ATP5IF1 would confer resistance to venetoclax. To test this, we used lentiviral particles expressing short hairpin RNA (shRNA) targeted to ATP5IF1 to generate ATP5IF1 knockdown AML cells lines (“AML2_shIF1_” and “MV411_shIF1_”). Reduced expression of ATP5IF1 was confirmed via Western blot (Fig. 6A). To confirm enhanced F_1_-ATPase activity in ATP5IF1 knockdown cells, we assessed the sensitivity of uncoupled respiration to inhibition by Δ*G*_ATP_. Compared to shRNA control cells, uncoupled respiration in ATP5IF1 knockdown cells was less sensitive to inhibition during the Δ*G*_ATP_ titration (Fig. 6B), consistent with enhanced F_1_-ATPase activity. When ATPase activity was directly measured in mitochondrial lysates, ATPase activity was enhanced in MV411_shIF1_ cells (fig. S6A). Having validated our ATP5IF1 knockdown model, we wanted to determine the impact of ATP5IF1 knockdown on AML intact cell respiration, respiratory capacity, OxPhos capacity, and fractional OxPhos. ATP5IF1 knockdown did not result in overt differences in intact cell respiration, respiratory capacity, or OxPhos capacity (Fig. 6, C to E, and fig. S6, B and C). However, fractional OxPhos was increased in AML2_shIF1_ cells (Fig. 6F). To confirm that F_1_-ATPase activity contributes to polarization in living ATP5IF1 knockdown AML cells, intact cell ΔΨ_m_ was assessed. Oligomycin triggered depolarization in MV411_shIF1_, indicating that living ATP5IF1 knockdown cells consume ATP (Fig. 6G). After characterizing the mitochondrial phenotype of ATP5IF1 knockdown AML cells, we tested the hypothesis that ATP5IF1 knockdown would confer resistance to venetoclax in AML cells. To test this, we measured the impact of ATP5IF1 knockdown on AML cell viability and colony formation in response to venetoclax. Relative to scrambled shRNA control cells, ATP5IF1 knockdown cells opposed reductions in cell viability and colony formation in response to venetoclax (Fig. 6, H to J). Collectively, these data demonstrate that knockdown of ATP5IF1 boosts matrix F1-ATPase activity, sufficient to confer partial resistance to venetoclax.

### Overexpression of ATP5IF1 blunts matrix ATP consumption and enhances sensitivity to venetoclax

**Fig. 7.**
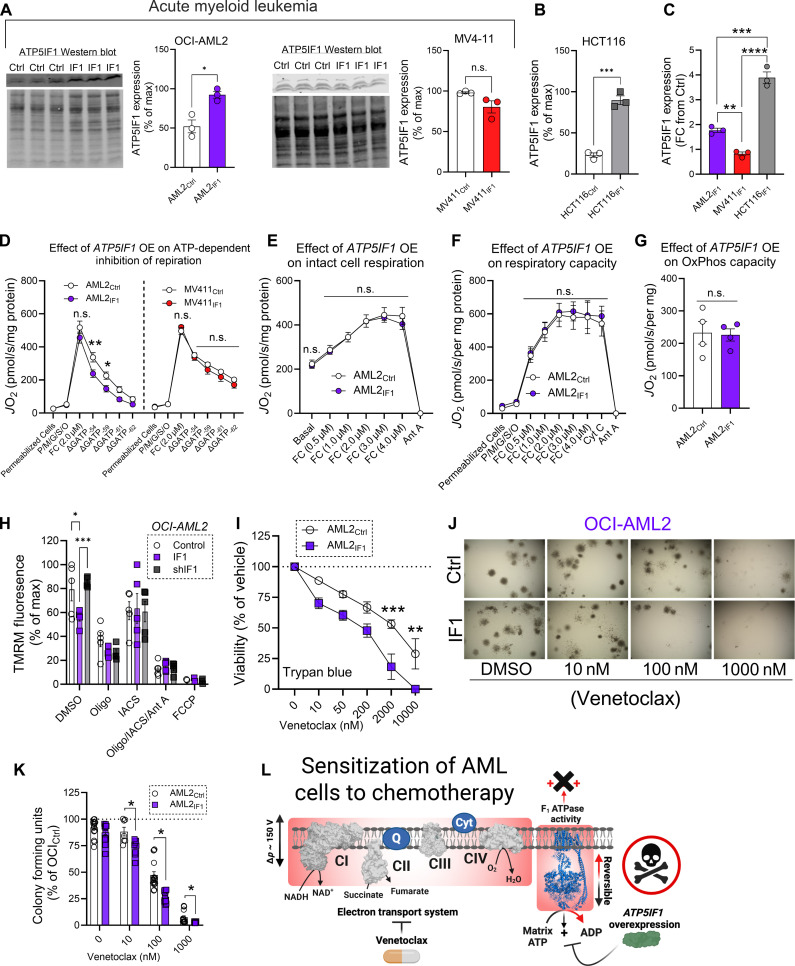
Overexpression of ATP5IF1 enhances sensitivity to BCL-2 targeted therapy. (**A**) Expression of ATP5IF1 in isolated mitochondria (*n* = 3 replicates). (**B**) Expression of ATP5IF1 in isolated mitochondria derived from lentivirus control HCT116 cells and ATP5IF1-overexpressing HCT116 cells (*n* = 3 replicates). (**C**) ATP5IF1 expression represented as fold change (FC) from lentivirus control AML and HCT116 cells (*n* = 3 replicates). (**D**) Impact of ATP5IF1 overexpression on the inhibition of respiration by Δ*G*_ATP_ in permeabilized lentivirus control AML cells and permeabilized ATP5IF1-overexpressing AML cells (*n* = 3 to 13 replicates). (**E**) Impact of ATP5IF1 overexpression on intact cell respiration in AML2_Ctrl_ cells and AML2_IF1_ cells (*n* = 5 replicates). (**F**) Impact of ATP5IF1 overexpression on respiratory capacity in permeabilized AML2_Ctrl_ cells and permeabilized AML2_IF1_ cells (*n* = 4 replicates). (**G**) Impact of ATP5IF1 overexpression on OxPhos capacity in permeabilized AML2_Ctrl_ cells and permeabilized AML2_IF1_ cells. (**H**) Flow cytometric analysis of ΔΨ_m_ using TMRM fluorescence in AML2_Ctrl_, AML2_shIF1_, and AML2_IF1_. Data expressed as a percentage of maximal TMRM fluorescence across all conditions (*n* = 5 replicates). (**I**) Effect of venetoclax on viability of AML2_Ctrl_ cells and AML2_IF1_ cells. Viability measured using trypan blue viable cell count (*n* = 5 replicates). (**J** to **K**) Effect of venetoclax on colony formation of AML2_Ctrl_ cells and AML2_IF1_ cells. Colony formation measured using CellTiter-Glo (*n* = 7 to 16 replicates). Representative image of colony formation of AML2_Ctrl_ cells and AML2_IF1_ cells in the presence or absence of venetoclax depicted in (J). (**L**) Schematic depicting mechanism of death of ATP5IF1-overexpressing AML cells in response to venetoclax. Created using BioRender.com. Data are presented as means ± SEM and analyzed by two-way ANOVA [(D) to (F) and (H)], one-way ANOVA (C), or unpaired *t* test [(A), (B), (G), (I), and (K)]. *P < 0.05; **P < 0.01; ***P < 0.001; ****P < 0.0001.

We hypothesized that overexpression of ATP5IF1 would reduce matrix ATP consumption and enhance venetoclax chemosensitivity. To test this, we used lentiviral particles containing an ATP5IF1 overexpression construct to attempt to generate AML cell lines with ATP5IF1 overexpression (AML2_IF1_, MV411_IF1_, and MM6_IF1_). In these models, ATP5IF1 expression is driven by the EF-1 alpha (Ef1a) promoter. For control, cells were also infected with lentiviral particles driving expression of the Cox8a mitochondrial targeting sequence tagged to the zsGreen1 fluorescent protein. After puromycin selection to establish stable cell lines, quantitative polymerase chain reaction (PCR) and Western blotting were performed to validate ATP5IF1 expression. In AML2_IF1_ cells, ATP5IF1 mRNA and protein levels were increased by threefold and twofold, respectively (Fig. 7A and fig. S6D). In contrast, no increase in ATP5IF1 mRNA or protein was observed in MV411_IF1_ cells (Fig. 7A and fig. S6D). In MM6_IF1_ cells, although there was a slight increase in ATP5IF1 mRNA, protein expression did not correspondingly increase (fig. S6, D and E). Despite the successful establishment of puromycin-resistant cell lines, the expected increase in ATP5IF1 expression was not observed in two of the three AML cell lines. Although it is possible that the ATP5IF1 expression vector was nonfunctional in these cells, we also considered the possibility that the transcription and/or the translation of the Ef1a-driven ATP5IF1 was being actively repressed. Previous studies have shown that ρ^0^ cells, which survive by maintaining mitochondrial polarization by consuming ATP, resist up-regulations in ATP5IF1 after infection with an ATP5IF1 overexpression virus (*34*). Because AML cells rely on matrix ATP hydrolysis to sustain ΔΨ_m_, we hypothesized that ATP5IF1 driven by the Ef1a promoter may be selectively silenced during the process of lentiviral infection and subsequent establishment of stable cell lines using puromycin. To test this hypothesis, we examined whether non-AML cells (HCT116), which do not exhibit matrix ATP hydrolysis, would show more robust ATP5IF1 overexpression when infected with the same lentiviral vector used in our AML models. In HCT116_IF1_ cells, ATP5IF1 expression increased nearly fourfold, surpassing the levels observed in the AML cell lines (Fig. 7, B and C, and fig. S6F).

Moving forward with the OCI-AML2 cell model, we next sought to determine whether ATP5IF1 overexpression was sufficient to affect F1-ATPase activity and cellular bioenergetics. To assess this, we first examined the sensitivity of uncoupled respiration to Δ*G*_ATP_ titration in permeabilized cells. Compared to AML2_Ctrl_, respiration in AML2_IF1_ cells was more strongly inhibited by ATP during the Δ*G*_ATP_ titration (Fig. 7D), consistent with an impaired ability to clear the ATP from the matrix. No differences in ATP sensitivity were observed between MV411_Ctrl_ and MV411_IF1_ cells (Fig. 7D), which is consistent with the lack of increase in ATP5IF1 expression in MV411_IF1_ cells. Direct measurement of ATPase activity in mitochondrial lysates from AML2_IF1_ cells confirmed decreased ATPase activity (fig. S6G). Next, we examined the effects of ATP5IF1 overexpression on intact cell respiration, respiratory capacity, OxPhos capacity, and mitochondrial membrane potential. No differences in respiration, respiratory capacity, or OxPhos capacity were observed between ATP5IF1-overexpressing cells and controls (Fig. 7, E to G, and fig. S6H). However, mitochondrial membrane potential was depolarized under basal conditions in AML2_IF1_ cells (Fig. 7H). Partial depolarization of ΔΨ_m_ combined with no change in OxPhos capacity and fractional OxPhos suggests that forced expression of ATP5IF1 specifically disrupts matrix F1-ATPase activity sufficient to blunt mitochondrial ATP consumption. To determine whether manipulation of mitochondrial ATP consumption was sufficient to alter cellular energy charge, phosphorylation of AMP-activated protein kinase (AMPK) was assessed in our ATP5IF1 gain-of-function and loss-of-function models. Phosphorylated AMPK relative to total AMPK was unaffected by ATP5IF1 expression (fig. S6I).

Having established the bioenergetic consequences of ATP5IF1 overexpression, we tested the hypothesis that this overexpression would sensitize AML cells to venetoclax. To assess this, we measured the effects of ATP5IF1 overexpression on AML cell viability and colony formation in response to venetoclax. AML2_IF1_ cells were more sensitive to reductions in cell viability and colony formation after venetoclax treatment compared to AML2_Ctrl_ cells (Fig. 7, I to K). In contrast, MM6_IF1_ cells, where ATP5IF1 expression was not increased, showed no change in sensitivity to venetoclax (fig. S6J). Collectively, these results support a model in which ATP5IF1 overexpression compromises AML cell viability in response to stress, highlighting matrix ATP consumption as a cancer cell–specific bioenergetic vulnerability, particularly relevant in the context of BCL-2-targeted chemotherapy (Fig. 7L).

## DISCUSSION

A diagnosis of AML is abysmal, as more than 70% of patients suffering from the disease will succumb to relapse (*55*). Relapse in AML is caused by the inability of current treatments to eradicate drug-resistant subclones that evade chemotherapy, fester below minimal residual disease cutoffs, and ultimately emerge to initiate fatal AML relapse (*56*). Therefore, improving AML patient outcomes depends on defining and ultimately thwarting the cellular mechanisms that drive chemotherapy resistance. Our prior research linked OxPhos deficiency to AML biology (*23*, *25*). The current investigation sought to (i) investigate bioenergetic mechanisms that underlie OxPhos deficiency, (ii) determine the link between OxPhos deficiency and AML chemoresistant disease, and (iii) identify AML-specific mitochondrial drug targets actionable in the context of chemoresistance.

Impaired AML OxPhos flux appears linked to lesions in the oxygen-consuming complex IV of the ETS. Herein, complex IV lesions were masked by increased mitochondrial content in AML cells. Only when respiratory capacity was normalized for mitochondrial content was it clear that maximal respiration intrinsic to each AML mitochondrial unit was lower. These findings agree with prior research in patient AML cells where it was discovered that the specific activity (i.e., flux per expression) of the terminal proton-pumping respiratory complexes (complexes III and IV) was decreased in AML (*6*). Lower respiration resulting from Complex IV lesions would be expected to hamper the ability of the ETS to generate a ΔΨ_m_ sufficient to fuel ATP synthesis. Consistent with this, when OxPhos was assessed in living AML cells, noncanonical ATP hydrolysis by the F_1_-ATPase contributed to polarization under basal conditions. Thus, although living AML cells do respire, they appear to simultaneously operate the ATP synthase reaction in reverse. The consumption of ATP by the AML F_1_-ATPase appears necessary to both support inner membrane polarization and sustain ETS function, as matrix ATP directly disrupts ETS flux, presumably via phosphorylation or allosteric regulation of matrix enzymes (*57*–*60*). ATP-dependent inhibition of ETS flux, combined with complex IV lesions, weakens the voltage potential across the AML inner membrane. Because ΔΨ_m_ drives matrix to cytosol ATP transport via ANT, a weakened voltage potential makes AML cells inherently reliant on F_1_-ATPase activity to (i) sustain polarization and (ii) facilitate the clearance of matrix ATP.

Regarding the source of ATP being consumed by the F_1_-ATPase, inhibition of glycolysis by 2-DG and inhibition of ANT by BA did not affect ΔΨ_m_. These findings suggest that the predominant source of ATP fueling F_1_-ATPase activity likely originates in the matrix space. Although our data demonstrate that ATP synthase hydrolyzes ATP in AML mitochondria, we cannot rule out the possibility that AML mitochondria are also capable of coupling respiration to the synthesis of ATP. That said, the preponderance of evidence suggests that any ATP made at ATP synthase is likely degraded, along with other matrix ATP sources, in support of sustaining ΔΨ_m_. Such a model is consistent with the reversal potentials of ANT relative to ATP synthase, which allow for a weakened ΔΨ_m_ generated by the ETS to allow ATP synthase to run in reverse without catalyzing ADP/ATP exchange via ANT (*30*). The lack of effect of ANT inhibition on ΔΨ_m_ and AML cell viability suggests that ANT minimally functions in AML cell under basal conditions. We propose that the interplay between complex IV lesions, matrix ATP, and F_1_-ATPase activity poise the AML mitochondrion to participate in a futile energy cycle where ATP is both made and degraded without considerable exchange with the cytosol. By degrading matrix ATP via F_1_-ATPase activity, AML cells oppose the inhibition of respiration by matrix ATP and simultaneously sustain polarization, thereby thwarting intrinsic apoptotic signaling. In doing so, the survival of AML cells is conferred despite intrinsic lesions in the proton pumping respiratory complexes and at the expense of the “healthy” communication between cellular Δ*G*_ATP_ demand in the cytosol and mitochondrial metabolism (*61*, *62*). Consistent with the lack of communication between the matrix and cytosol, manipulation of matrix ATP hydrolysis via ATP5IF1 knockdown and/or overexpression did not affect cellular energy charge, as assessed by phosphorylated AMPK. Given that oxidative metabolism in AML mitochondria appears to be, at least, in part, supported by the degradation of matrix ATP via the F_1_-ATPase, we have termed this unique bioenergetic pathway “dephosphorylative oxidation” (i.e., “DephOx”), as opposed to canonical “OxPhos.”

The ability of AML mitochondria to both respire and consume ATP via the F_1_-ATPase provides two distinct sources of polarization across the inner membrane, the ETS and F_1_-ATPase. As a result, F_1_-ATPase activity can sustain polarization during respiratory inhibition and thwart apoptotic signaling. In agreement with this, AML cells sustained polarization in response to IACS and antimycin A by consuming ATP via the F_1_-ATPase. Polarization via F_1_-ATPase activity makes AML cells resistant to apoptosis in response to both IACS and antimycin A. Although respiratory inhibitors have demonstrated promising preclinical efficacy against AML (*11*, *51*, *63*), the lack of translational efficacy (*16*, *64*) in human trials can be explained, in part, by the inability of these therapies to disrupt polarization originating from the F_1_-ATPase. Related to this, the chemotherapeutic venetoclax is also known to inhibit AML mitochondrial respiration (*37*, *42*, *43*). AML cells exposed to venetoclax presented with a collapse in mitochondrial respiration that tracked with an inhibition of polarization originating from the ETS. Despite incurring mitochondrial damage and a collapse in mitochondrial respiration, living AML cells exposed to venetoclax remained polarized by consuming ATP via the F_1_-ATPase. Disrupting F_1_-ATPase activity with oligomycin in combination with venetoclax resulted in enhanced toxicity, revealing polarization via the F_1_-ATPase as crucial for AML survival during BCL-2 inhibition. Consistent with this idea, venetoclax retained its bioenergetic impact on mitochondrial respiration in drug-resistant AML. These data suggest that continual venetoclax pressure applied in combination with an agent that specifically disrupts polarization by the F_1_-ATPase may accelerate AML cell death in cells otherwise resistant to the cytotoxic effects of venetoclax. Previous reports have demonstrated that AML cell survival is reliant on the activity of the F_1_-ATPase/ATP synthase (*65*). Discovered to bind and inhibit ATP synthase at the F_1_ subunit, ammocidin A (i.e., a macrolide) achieved AML cell toxicity in vivo with minimal organ toxicity (*65*). The minimal organ toxicity by ammocidin A suggests the possibility that ammocidin A may preferentially inhibit ATP hydrolysis by the F_1_-ATPase, with potential to restore chemosensitivity akin to an ATP5IF1 mimetic. In our hands, when ATP5IF1 expression was knocked down in AML cells to enhance F_1_-ATPase activity, resistance to venetoclax was conferred. Conversely, chemosensitivity to venetoclax was enhanced when F_1_-ATPase activity was blunted by overexpression of ATP5IF1 in OCI-AML2 cells.

In the present study, ΔΨ_m_ sustained by ATP hydrolysis was inferred on the basis of a depolarization effect observed following exposure to oligomycin. Although we feel that this method is the best available biochemical technique to infer the direction of ATP synthase in free living cells, the lack of direct measurement of ATP synthase working in reverse in living AML cells is a limitation of the study. While ATP synthase reversal to maintain ΔΨ_m_ is well established under conditions of severe respiratory inhibition (*34*), recent findings support our conclusion that ATP hydrolysis can also occur under physiological conditions, where both respiration and matrix ATP hydrolysis happen simultaneously (*66*). In this work, thermogenic brown adipose tissue was shown to rely on ATP hydrolysis to support maximal uncoupled respiration in thermogenic brown fat mitochondria (*66*), demonstrating that matrix ATP hydrolysis can coexist with ETS flux provided that the ΔΨ_m_ is partially depolarized to allow ATP synthase reversal. This condition is likely similar to what we observe in AML, where minimal ΔΨ_m_ generated by the ETS coexists with matrix ATP hydrolysis. However, we cannot definitively identify the exact mechanism(s) of mitochondrial depolarization in response to oligomycin. It is possible that oligomycin both inhibits ΔΨ_m_ generated by ATP synthase while also allowing accumulated matrix ATP to dampen respiration, thereby also reducing ΔΨ_m_ generated by the ETS. We present evidence for both mechanisms in the current study.

Collectively, our data demonstrate that AML cells resist chemotherapy-induced apoptosis by hydrolyzing ATP via the F_1_-ATPase to sustain polarization. Polarization of ΔΨ_m_ via F_1_-ATPase activity is generally observed to be a feature of bioenergetically “sick” cells. Given our extensive data revealing AML mitochondria to be bioenergetically sick, targeting F_1_-ATPase activity in AML cells represents an exciting opportunity to intervene on a unique AML-specific mitochondrial pathway.

## MATERIALS AND METHODS

All procedures involving human subjects were approved by the Institutional Review Board of the Brody School of Medicine (study IDs: UMCIRB 18–001328 and UMCIRB 19–002331) and Wake Forest University School of Medicine (study ID: IRB00117251). All samples were collected in accordance with the Declaration of Helsinki and with informed consent. All procedures on experimental animals were approved by the East Carolina University Institutional Animal Care and Use Committee (animal use protocol numbers Q362 and Q365).

### Blood collection and isolation of PBMCs

Venous blood from the brachial region of the upper arm was collected from healthy volunteers, between the ages of 18 and 70 years. Whole blood was collected in sodium-heparinized Cell Preparation Tubes (CPTs) (BD Biosciences, Franklin Lakes, NJ) and was then centrifuged at 1800*g* for 15 min. Mononuclear cells were washed in ammonium-chloride-potassium (ACK) lysis buffer to remove red blood cells, and PBMCs were either used immediately or banked in vapor phase of liquid nitrogen.

### Mononuclear cell isolation from bone marrow aspirates

For primary leukemia samples, bone marrow aspirates were collected from patients undergoing confirmatory diagnosis for a range of hematological malignancies. Patients with confirmed AML were enrolled in the study. All patients provided informed consent before study enrollment (study ID: UMCIRB 19–002331). Patient age ranged from 32 to 78 years. Bone marrow aspirates were collected in sodium-heparinized CPTs (BD Biosciences, no. 362761, Franklin Lakes, NJ) and centrifuged at 1800*g* for 15 min. Mononuclear cells were isolated and then washed in ACK lysis buffer to remove red blood cells and used immediately for experiments or banked in vapor phase of liquid nitrogen before experimentation. For healthy controls, bone marrow aspirates were collected from healthy donors, ages 26 to 33, supplied by HemaCare (Northridge, CA). Additional heathy bone marrow aspirates were derived from patients’ samples that were discovered to not have a hematological cancer. CD34^+^ cells were purchased from HemaCare (Northridge, CA).

### Culturing of AML cell lines

MV411 [American Type Culture Collection (ATCC), no. CRL-9591], HL-60 (ATCC, no. CCL-240), and KG-1 (ATCC, no. CCL-246) were originally purchased from ATCC, Manassas, VA. OCI-AML2 (often abbreviated throughout as “AML2”; DSMZ, no. ACC99) cells were a gift from M. Minden (Princess Margaret Cancer Center). The Mono Mac 6 (MM6; DSMZ, no. ACC124) cells were used in accordance with a signed material transfer agreement. MOLM-13-YFP-Luc cells (labeled throughout paper as MOLM-13 or MOLM-13_WT_; DSMZ, no. ACC554) were gifted by H.-G. Wang (Penn State University, Hershey, PA). THP-1 cells (ATCC, no. TIB-202) were given by K. Doi and H.-G. Wang from Penn State College of Medicine. OCI-AML3 (DSMZ, no. ACC582) cells were gifted from X. Gu, Cleveland Clinic. AML cell lines were cultured in either IMDM (Thermo Fisher Scientific, 31980030) or RPMI 1640 (Thermo Fisher Scientific, 61870-036) supplemented with 10% fetal bovine serum (FBS; Peak Serum, no. PS-FB1) and 1% penicillin/streptomycin (Gibco, no. 15140) and incubated at 37°C in 5% CO_2_. Cells were used for experimentation after reaching an average density 1.0 × 10^6^ cells/ml, and fresh medium was provided 24 hours before harvesting.

For experiments involving venetoclax resistance, MV411, OCI-AML-2, and MOLM-13 cells were cultured in IMDM (Thermo Fisher Scientific, 31980-030) or RPMI 1640 (Thermo Fisher Scientific, 61870-036), supplemented with 10% FBS and 1% penicillin/streptomycin. To model venetoclax resistance, cells were adapted in culture to 1 μM venetoclax (Selleck Chemicals, S8048). To do so, AML cells were serially passaged in increasing doses of venetoclax, beginning at 2 nM and doubling until reaching a final concentration of 1 μM. Venetoclax concentration was doubled every 2 to 3 weeks (~2 passages per week) until a final concentration of 1 μM was reached. While the time required to establish each cell line varied, the average duration for completion was 16 to 18 weeks. Venetoclax-resistant AML cells were continually maintained in 1 μM venetoclax unless otherwise indicated. Cells were used for experimentation after reaching an average density 1.0 × 10^6^ cells/ml, and fresh medium was provided 24 hours before harvesting.

For experiments involving respiration-deficient OCI-AML2 ρ^0^ cells, ρ^0^ were generated by passaging OCI-AML2 in IMDM (Thermo Fisher Scientific, 31980-030), supplemented with 10% FBS, 1% penicillin/streptomycin, ethidium bromide (50 ng/ml), pyruvate (100 μg/ml), and uridine (100 μg/ml) until a lack of basal respiration was confirmed, similar to that described previously (*46*). Cells were used for experimentation after reaching an average density 1.0 × 10^6^ cells/ml, and fresh medium was provided 24 hours before harvesting.

### Cell viability

Cells were centrifuged at 300*g* for 7 min, and the culture medium was aspirated. Cells were then washed in phosphate-buffered saline (PBS) and centrifuged again at 300*g* for 7 min. PBS was then aspirated, and cells were seeded in either IMDM (Thermo Fisher Scientific, 31980-030) or RPMI 1640 (Thermo Fisher Scientific, 61870-036), supplemented with 10% FBS and 1% penicillin/streptomycin at an average density of 0.2 × 10^6^ cells/ml. Cell suspension was then loaded into 12-well plates at a volume of 0.5 ml, and the indicated agents were added to each well relative to a vehicle control. After addition of agents, cells were incubated at 37°C and 5% CO_2_ for 48 hours. At the end of the 48 hours, cell viability was measured using trypan blue (0.4%) (Thermo Fisher Scientific, 15250-061) viable cell count. Where otherwise indicated, viability was determined by fluorescence measurement using propidium iodide (PI) (Thermo Fisher Scientific, P3566) or luminescence measurement using CellTiter-Glo (Promega, G968A).

For fluorescent measurements of cell viability using PI, cells were seeded in black-wall, 96-well plates, in growth medium. Water was used as a blank to subtract background fluorescence. After addition of agents (0.2 ml of final well volume), cells were incubated at 37°C and 5% CO_2_ for 48 hours. After 48 hours of incubation, positive control cells (used to indicate 100% death) were first permeabilized by addition of 1 μl of digitonin (10.0 mg/ml) and incubated at 37°C and 5% CO_2_ for 20 min. Following this, the plates were centrifuged at 1200*g* for 10 min. After centrifugation, plates were dumped to remove culture medium, and 0.1 ml of a 5.0 μM PI solution in PBS was added to each well. The plate was then incubated at 37°C and 5% CO_2_ for 20 min, and total death (%) was calculated using the ratio of the fluorescence signal of the treatment group and the positive control at 530-nm excitation and 620-nm emission. To determine viability, the total death (%) was subtracted from 100 and normalized relative to the vehicle control. For all viability assays using PI, each biological replicate was derived from the mean of two technical replicates. For calculation of Bliss Synergy score, Synergy Finder Plus was used, with % viability as the readout (*67*).

### Colony formation and clonogenicity

Colony formation and clonogenicity of AML cells was performed using human methylcellulose complete medium (Bio-Techne, 390395). To begin, AML cells were harvested and centrifuged at 300*g* for 7 min. Following this, AML cells were resuspended at an average density of 1 × 10^4^ cells/ml to make a 10× stock. The 10× stock of AML cells was then added to separate tubes containing human methylcellulose complete medium to make a 1× cell suspension, and each tube was supplemented with the inhibitors/agents indicated in the figure legends (e.g., venetoclax). Next, 300 μl of each cell suspension was seeded in 48-well plate, and any empty wells were filled with sterile and deionized H_2_O to prevent dehydration of the medium. The plate was then incubated at 37°C and 5% CO_2_ for 7 to 10 days. At the end of the incubation period, colony formation was measured by counting colonies using microscopy or via CellTiter-Glo (Promega, G968A). Representative images were captured using an EVOS FL Auto Imaging System (Thermo Fisher Scientific).

### Quantification and normalization of respiratory flux

Respirometry measurements were conducted using an Oroboros Oxgraph-2k (O2k; Oroboros Instruments, Innsbruck, Austria) in either a 0.5- or 1.0-ml reaction volume at 37°C. Data were normalized to viable cell count using trypan blue (0.4%) (Thermo Fisher Scientific, 15250-061), unless otherwise indicated. For normalization to total protein, cell suspensions were removed from the O2k chamber following the completion of each assay and placed into microcentrifuge tubes. The cell suspension was then centrifuged at 2000*g* for 10 min at 4°C. Assay buffer was aspirated, and the cell pellet was washed with PBS. Cells were then lysed using low-percentage detergent buffer, CelLytic M (Sigma-Aldrich, C2978), followed by a freeze-thaw cycle on dry ice. The protein concentration was then determined using a Pierce bicinchoninic acid (BCA) protein assay (Sigma-Aldrich, 71285).

### Intact cell respiration

To assess intact cell respiration, ~1 × 10^6^ to 2 × 10^6^ cells were harvested and centrifuged at 300*g*. After centrifugation, cells were washed in PBS and centrifuged again at 300*g*. After this, PBS was aspirated and cells were resuspended in either 0.5 or 1 ml of intact cell respiratory medium (bicarbonate-free IMDM, pH set to 7.4 with Hepes and supplemented with 1% penicillin/streptomycin and 10% FBS). Viable cell count was then performed with trypan blue (0.4%) (Thermo Fisher Scientific, 15250-061). Basal respiration was then quantified, followed by FCCP titration (FC; 0.5 to 5 μM). Antimycin A (Ant A; 0.5 μM) was then added as a negative control to inhibit mitochondrial respiration.

### Permeabilized cell respiratory capacity

To measure respiratory capacity, ~1 × 10^6^ to 2 × 10^6^ cells were harvested and centrifuged at 300*g*. After centrifugation, cells were washed in PBS and centrifuged again at 300*g*. Cells were then resuspended in either 0.5 or 1 ml of Respiratory Buffer supplemented with creatine [CR; 105 mM MES potassium salt, 30 mM KCl, 8 mM NaCl, 1 mM EGTA, 10 mM KH_2_PO_4_, 5 mM MgCl_2_, 0.25% bovine serum albumin (BSA), and 5 mM CR monohydrate (pH 7.2)]. Viable cell count was then performed with trypan blue (0.4%) (Thermo Fisher Scientific, 15250-061). Cells were then permeabilized with digitonin (0.02 mg/ml). Respiratory capacity was then assessed by energizing the mitochondria with the indicated carbon substrates, either pyruvate (Pyr or P; 5 mM), malate (M; 1 mM), octanoyl-carnitine (O; 0.2 mM), succinate (Succ or S; 5 mM), glutamate (Glut or G; 5 mM), or combinations thereof, followed by FCCP titration (FC; 0.5 to 5 μM). Antimycin A (Ant A; 0.5 μM) was then added as a negative control to inhibit mitochondrial respiration. Respiratory capacity was determined by the maximal respiration stimulated by any dose of FCCP across the FCCP titration.

### OxPhos respiratory kinetics and OxPhos capacity

OxPhos respiratory kinetics were measured using a modified version of the CR-kinase clamp (CK clamp). For experiments using the CK clamp assay, the free energy of ATP hydrolysis (Δ*G*_ATP_) is calculated using the equilibrium constant for the CK reaction (*K′*_CK_) and is based upon the addition of known concentrations of CR, phosphocreatine (PCr), and ATP in the presence of excess amounts of CK. Calculation of Δ*G*_ATP_ at defined PCr concentrations was done using the online resource (https://dmpio.github.io/bioenergetic-calculators/ck_clamp/) as previously described (*68*). For all assays using the CK clamp technique, various combinations of carbon substrates and inhibitors were used.

To begin to assess OxPhos respiratory kinetics, ~1 × 10^6^ to 2 × 10^6^ cells were harvested and centrifuged at 300*g*. After centrifugation, cells were washed in PBS and centrifuged again at 300*g*. Cells were then resuspended in either 0.5 or 1 ml of Respiratory Buffer supplemented with CR [105 mM MES potassium salt, 30 mM KCl, 8 mM NaCl, 1 mM EGTA, 10 mM KH_2_PO_4_, 5 mM MgCl_2_, 0.25% BSA, and 5 mM CR monohydrate (pH 7.2)]. Viable cell count was then performed with trypan blue (0.4%) (Thermo Fisher Scientific, 15250-061). Cells were then permeabilized with digitonin (0.02 mg/ml). OxPhos respiratory kinetics were then assessed by energizing the mitochondria with the indicated carbon substrates, either pyruvate (Pyr or P; 5 mM), malate (M; 1 mM), octanoyl-carnitine (O; 0.2 mM), succinate (Succ or S; 5 mM), glutamate (Glut or G; 5 mM), or combinations thereof. OxPhos respiratory kinetics were then quantified across a span of physiologically relevant Δ*G*_ATP_ values (ranging from −54.2 to −61.5 kJ/mol) using the CK clamp [CK (20 U/ml), ATP (5 mM), and PCr (1, 6, 15, and 21 mM)]. At the end of the Δ*G*_ATP_ titration, oligomycin was added to inhibit ATP synthase, unless otherwise indicated. Following this, maximal respiration was quantified using FCCP uncoupler. At the end of the assay, unless otherwise indicated, antimycin A (Ant A; 0.5 μM) was used as a negative control to inhibit mitochondrial respiration. Unless otherwise indicated, the OxPhos capacity of each cell line was calculated as the maximal respiration stimulated by a Δ*G*_ATP_ value of −54.2 kJ/mol, supported by saturating carbon substrates (P/M/G/S/O). Adaptations of the OxPhos kinetics assay were used to quantify OxPhos capacity supported by either complex I (P/M/G)– or complex II (S)–linked substrates.

Other substrates and inhibitors used in each assay are indicated in the figure legends: CK (20 U/ml), ATP (5 mM), PCr (1, 6, 15, and 21 mM), pyruvate (Pyr or P; 5 mM), malate (M; 1 mM), octanoyl-carnitine (O; 0.2 mM), succinate (Succ or S; 5 mM), glutamate (Glut or G; 5 mM), oligomycin (Oligo; 0.02 μM), FCCP (0.5 to 2 μM), rotenone (Rot or R; 0.5 μM), antimycin A (Ant A; 0.5 μM), venetoclax (Vclax; 0.1 nM-1 μM), FCCP (0.5 to 6 μM), CAT (5 μM), BA (25 to 50 μM), and alamethicin (0.03 mg/ml).

### Isolation of mitochondria from AML cells

Starting material for all isolations was between 300 and 400 cells. Cells were harvested and centrifuged at 300*g* for 7 min, washed in ice cold PBS, and centrifuged again at 500*g* for 10 min. Cell pellets were then resuspended in Mitochondrial Isolation Buffer with BSA [100 mM KCl, 50 mM Mops, 1 mM EGTA, 5 mM MgSO_4_, and 0.2% BSA (pH 7.1)] and homogenized using a borosilicate glass mortar and Teflon pestle. Cell homogenate was centrifuged at 800*g* for 10 min at 4°C. Following this, supernatant was collected and centrifuged at 10,000*g* for 10 min at 4°C to pellet the mitochondrial fraction. The mitochondrial fraction was resuspended in Mitochondrial Isolation Buffer without BSA, transferred to a microcentrifuge tube, and centrifuged again at 10,000*g*. The mitochondrial pellet was then resuspended in ~200 μl of Mitochondrial Isolation Buffer, and protein was quantified using the Pierce BCA assay.

Respiration assays using isolated mitochondria were similar to that described for permeabilized cells. For experiments using permeabilized mitochondria, alamethicin (0.03 mg/ml) was used for permeabilization. In addition to reagents used in the permeabilized cell assays, NADH (4 mM) was used to assess complex I–supported respiration in alamethicin-permeabilized mitochondria experiments.

### Isolation of mitochondria from mouse heart

Hearts were derived from C57BL/6J mice (the Jackson Laboratory, strain no. 000664). Heart was dissected and placed in ice-cold Mitochondrial Isolation Buffer with BSA [100 mM KCl, 50 mM Mops, 1 mM EGTA, 5 mM MgSO_4_, and 0.2% BSA (pH 7.1)]. Tissue was then minced, resuspended in 10 ml of Mitochondrial Isolation Buffer and homogenized for ~10 passes via a Teflon pestle and borosilicate glass vessel. The homogenate was transferred to a 15-ml tube, the volume was brought to 12 ml, and sample was centrifuged at 800*g* for 10 min at 4°C. The supernatant was transferred to a fresh 15-ml tube and spun at 10,000*g* for 10 min at 4°C. Mitochondrial pellet was washed in 1.4 ml of Mitochondrial Isolation Buffer without BSA [100 mM KCl, 50 mM Mops, 1 mM EGTA, and 5 mM MgSO_4_ (pH 7.1)] and spun at 10,000*g* for 10 min at 4°C. The resulting pellet was resuspended in Mitochondrial Isolation Buffer without BSA. Protein content was determined using the Pierce BCA assay.

### Assessment of mitochondrial membrane potential in whole intact cells using flow cytometry

Cells were harvested and resuspended in IMDM or RPMI 1640 growth medium at a density of ~1 × 10^6^ cells/ml. After this, cells were treated with either oligomycin (5 μM), rotenone (0.5 μM), antimycin A (0.5 μM), BA (50 μM), venetoclax (10 to 1000 nM), CB-839 (5 μM) (MedChemExpress, no. HY-12248), CPI-613 (200 μM) (MedChemExpress, no. HY-15453), or 2-DG (50 mM), where indicated, for 15 min at 37°C. FCCP (10 μM) or alamethicin (0.03 mg/ml) was used as negative controls. The cells were stained with TMRM (10 to 20 nM; Thermo Fisher Scientific, no. T668) and incubated for 30 min at 37°C with 5% CO_2_. Thereafter, 4′,6-diamidino-2-phenylindole (DAPI; 1 μg/ml) was added. Samples were then analyzed with a Cytek Aurora flow cytometer. Data were analyzed by excluding cells determined to be nonviable based on DAPI staining. Given that TMRM is a functional dye, all viable cells were included during the quantification of TMRM fluorescence. FlowJo software v10.9.0 (Treestar, USA) was used for data analysis.

### Assessment of mitochondrial membrane potential in whole intact cells using fluorescence microscopy

Cells were harvested and resuspended in (Thermo Fisher Scientific, 31980-030) medium at a density of 1 × 10^6^ cells/ml. After this, cells were treated with either oligomycin (5 μM), rotenone (0.5 μM), antimycin A (0.5 μM), BA (20 μM), or venetoclax (10 to 1000 nM), where indicated, for 15 min at 37°C. FCCP (10 μM) or alamethicin (0.03 mg/ml) was used as negative controls. The cells were stained with TMRM (100 nM) and incubated for 30 min at 37°C with 5% CO_2_. Cells were imaged using fluorescence microscopy with an EVOS FL Auto Imaging System (Thermo Fisher Scientific). Quantification of TMRM fluorescence was performed using CellProfiler v4.2.5 (*69*). For quantification of TMRM fluorescence with CellProfiler, images taken with fluorescence microscopy were first uploaded into CellProfiler. Following this, the following modules were added to the pipeline for analyzing each image: (i) IdentifyPrimaryObjects, (ii) MeasureObjectIntensity, (iii) CalculateMath, and (iv) ExportToSpreadsheet. The specific parameters for each module (e.g., typical diameter of objects) were defined on a per-experiment basis to optimize the analysis for each image set. In general, cells on the border of the image were excluded from every analysis. The raw analysis was then exported to spreadsheet, and the TMRM fluorescence of each identified object (i.e., each individual cell) was normalized to the average TMRM fluorescence of the vehicle control after subtracting the fluorescence of either FCCP (10 μM) or alamethicin (0.03 mg/ml) from all identified objects. Following normalization, the TMRM fluorescence of each identified object in each analyzed image was plotted and represented as individual cells.

For confocal imaging, cells were suspended in IMDM formulation medium (Thermo Fisher Scientific) containing 50 nM TMRM and 2 μM Hoechst 33342. Cells were plated on glass-bottom dishes (MatTek, Ashland, MA) for imaging. Cells were held in place by pre-coating the glass bottom dishes with Cell-Tak (BD, 0.02 mg/ml in 0.1 μM NaHCO_3_) for 20 min. Cells were attached to the plate by centrifugation at 1000*g*, without brakes for 5 min. All imaging was performed using an Olympus FV1000 laser scanning confocal microscope with an onstage incubator at 37°C. Acquisition software was Olympus FluoView FSW (V4.2). The objective used was 60× oil immersion [numerical aperture of 1.35, Olympus Plan Apochromat UPLSAPO60X(F)]. Images were 800 × 800 pixel with dwell time of 2 μs/pixel and sequential scan mode, resulting in a 4× digital zoom. Hoechst 33342 was excited using the 405-nm line of a multiline argon laser; emission was filtered using a 560-nm dichroic mirror and 420- to 460-nm barrier filter. TMRM was excited using a 559-nm laser diode; emission was filtered using a 575- to 675-nm barrier filter. Zero detector offset was used for all images, and gain at the detectors was kept the same for all imaging. The pinhole aperture diameter was set to 105 μm (one Airy disc). Images were analyzed using Fiji (*70*). Spatial resolution was measured using sub-resolution fluorescent beads (Thermo Fisher Scientific), and curve fitting was performed using the MetroloJ plugin in Fiji. Sixteen-bit images were made into a composite. ΔΨ_m_ was measured using non-quenching concentrations of TMRM. Out-of-field images from the axial dimension were trimmed from each stack. Image stacks were then background corrected using dark-field images. Median background intensity (*I*_B_) was measured by inverting a Huang auto threshold for each image stack. The estimated electrical potential difference relative to the median value of the extramitochondrial signal was then mapped onto each pixel in each image using a math macro

$$∆\Psi_{m}\left(\text{mV}\right)=2.303*\frac{\mathrm{RT}}{\mathrm{zF}}*\text{log}\left(\frac{v}{I_{B}}\right)$$

where *R* is the universal gas constant (joules per mole per kelvin), *T* is the absolute temperature (310.15 K), *z* is the + 1 charge of TMRM, *F* is Faraday’s constant, and *v* is the gray value of the designated pixel. A Huang auto threshold was then applied to each image in the stack, and median and maximum values for each threshold were obtained. This measurement method reduced biasing of the total measured signal toward low-intensity pixel values.

### Assessment of membrane potential of isolated mitochondria using flow cytometry

Mitochondria isolated from mouse heart or HL60 cells were suspended in Respiratory Buffer supplemented with CR [105 mM MES potassium salt, 30 mM KCl, 8 mM NaCl, 1 mM EGTA, 10 mM KH_2_PO_4_, 5 mM MgCl_2_, 0.25% BSA, and 5 mM CR monohydrate (pH 7.2)] and then aliquoted into reaction tubes. Reactions tubes were treated with (i) H_2_O (vehicle); (ii) pyruvate (Pyr or P; 5 mM) and malate (M; 1 mM); (iii) P/M, CK (20 U/mL), ATP (5 mM), and PCr (21 mM); (iv) P/M, CK (20 U/ml), ATP (5 mM), PCr (21 mM), and Oligo (5 μM); or (v) P/M, CK (20 U/ml), ATP (5 mM), PCr (21 mM), Oligo (5 μM), and FCCP (5 μM) for 15 min at room temperature. Mitochondrial reaction tubes were then exposed to TMRM (10 nM) and incubated for 30 min at 37°C with 5% CO_2_. Samples were then analyzed with a Cytek Aurora flow cytometer. TMRM fluorescence intensity was quantified using FlowJo software v10.9.0 (Treestar, USA).

### Assessment of mitochondrial membrane potential in permeabilized cells

Mitochondrial membrane potential in permeabilized cells was performed using methods described previously with some adaptations (*68*). Fluorescent determination of the mitochondrial membrane potential was performed using a QuantaMaster Spectrofluorometer (QM-400; Horiba Scientific). Polarization was determined using TMRM fluorescence operating in quench mode, as described previously (*68*). Cells were harvested, centrifuged at 300*g* for 7 min, then washed with PBS, and centrifuged again at 300*g*. Following this, cells were resuspended in 1 ml of Respiratory Buffer supplemented with CR [105 mM MES potassium salt, 30 mM KCl, 8 mM NaCl, 1 mM EGTA, 10 mM KH_2_PO_4_, 5 mM MgCl_2_, 0.25% BSA, and 5 mM CR monohydrate (pH 7.2)]. Viable cell count was then performed with trypan blue (0.4%) (Thermo Fisher Scientific, 15250-061). Following this, cells were permeabilized with digitonin (Digi; 0.02 mg/ml) for 10 min at room temperature, with gentle rocking. From that permeabilized cell suspension, ~3 million cells were added to a microcentrifuge tube and centrifuged at 1500*g* for 10 min. The permeabilized cell pellet was resuspended in fresh Respiratory Buffer and loaded with TMRM (200 nM). Optimal excitation and emission parameters for each cell type were determined using an excitation/emission scan to determine the fluorescence ratio, as described previously (*68*). Membrane potential was then quantified using a fluorescence ratio of the experimentally determined excitation and emission parameters.

In general, permeabilized cell membrane potential assays were similar to those used in respiration assays. Substrates and inhibitors used in each assay are indicated in the figure legends: CK (20 U/mL), ATP (5 mM), PCr (1, 6, 15, and 21 mM), pyruvate (Pyr or P; 5 mM), malate (M; 1 mM), oligomycin (Oligo; 0.02 μM), FCCP (5 μM), rotenone (Rot or R; 5 to 100 nM), antimycin A (Ant A; 0.5 μM), venetoclax (Vclax; 0.1 nM to 1 μM), FCCP (5 μM), and CAT (5 μM).

### Assessment of ATP-dependent inhibition of respiration and polarization using permeabilized cells

To assess the inhibition of respiration by ATP, cells were harvested and centrifuged at 300*g*. After centrifugation, cells were washed in PBS and centrifuged again at 300*g*. Cells were then resuspended in either 0.5 or 1 ml of Respiratory Buffer supplemented with CR [105 mM MES potassium salt, 30 mM KCl, 8 mM NaCl, 1 mM EGTA, 10 mM KH_2_PO_4_, 5 mM MgCl_2_, 0.25% BSA, and 5 mM CR monohydrate (pH 7.2)]. Viable cell count was then performed with trypan blue (0.4%) (Thermo Fisher Scientific, 15250-061). Cells were then permeabilized with digitonin (0.02 mg/ml). Following permeabilization, cells were energized with saturating carbon substrates: pyruvate (Pyr or P; 5 mM), malate (M; 1 mM), octanoyl-carnitine (O; 0.2 mM), succinate (Succ or S; 5 mM), and glutamate (Glut or G; 5 mM). Uncoupled respiration was stimulated with FCCP (1.5 to 2 μM). In wild-type AML cells, oligomycin (0.02 μM) or no oligomycin was added to inhibit the reversal of ATP synthase. The inhibition of uncoupled respiration by ATP was then quantified across a span of physiologically relevant Δ*G*_ATP_ values (ranging from −54.2 to −61.5 kJ/mol) using the CK clamp [CK (20 U/ml), ATP (5 mM), and PCr (1, 6, 15, and 21 mM)]. When this assay involved cells that have undergone genetic manipulation of ATP5IF1 expression, oligomycin was not used.

To assess the inhibition of polarization by ATP, cells were harvested and centrifuged at 300*g*. After centrifugation, cells were washed in PBS and centrifuged again at 300*g*. Cells were then resuspended in either 0.5 or 1 ml of Respiratory Buffer supplemented with CR [05 mM MES potassium salt, 30 mM KCl, 8 mM NaCl, 1 mM EGTA, 10 mM KH_2_PO_4_, 5 mM MgCl_2_, 0.25% BSA, and 5 mM CR monohydrate (pH 7.2)]. Viable cell count was then performed with trypan blue (0.4%) (Thermo Fisher Scientific, 15250-061). Cells were then permeabilized with digitonin (0.02 mg/ml) and gently rocked for 10 min. From that permeabilized cell suspension, ~3 million cells were added to a microcentrifuge tube and centrifuged at 1500*g* for 10 min. The permeabilized cell pellet was resuspended in fresh Respiratory Buffer and loaded with TMRM (200 nM), and the membrane potential was assessed, similar to that described in the permeabilized cell membrane potential assay with some modifications. Oligomycin (0.02 μM) was added to inhibit ATP synthase, and either CAT (5 μM) or no CAT was added to inhibit the ANT. Mitochondria were then polarized with pyruvate (Pyr or P; 5 mM) and malate (M; 1 mM). The inhibition of polarization by ATP was then quantified across a span of physiologically relevant Δ*G*_ATP_ values (ranging from −54.2 to −61.5 kJ/mol) using the CK clamp [CK (20 U/ml), ATP (5 mM), and PCr (1, 6, 15, and 21 mM)]. Antimycin A (Ant A; 0.5 μM) was then added followed by FCCP (5 μM) as a negative control.

### Lentiviral ATP5IF1 knockdown in AML cells

OCI-AML2 or MV4-11 cells were cultured in IMDM (Thermo Fisher Scientific, 31980-030) supplemented with 10% FBS and 1% penicillin/streptomycin and incubated at 37°C in 5% CO_2_. Human shRNA lentiviral particles cloned into pLKO.1 vector (18-nucleotide oligomer ATP5IF1-specific shRNA 5′-GGCTGAAGAGGAACGATA-3′) and one scramble control [0.5 ml each, >10^7^ transduction units (TU)/ml] were purchased from Addgene (catalog no. 10878). To facilitate infection, AML cells and lentiviral particles were cocultured at a seeding density of 1 × 10^6^ cells/ml in a RetroNectin dish (Takara Bio, T110A). Following 72 hours of infection, cultures were then subjected to puromycin selection by continuous exposure to puromycin (1 to 2 μg/ml) in the culture medium.

To confirm knockdown of ATP5IF1 expression, mitochondria were first isolated from scrambled shRNA control cells and ATP5IF1 knockdown AML cells. Mitochondria were then lysed using Pierce RIPA (radioimmunoprecipitation assay) Buffer (Thermo Fisher Scientific, 89900) and were supplemented with 1× Halt Protease and Phosphatase Inhibitor Cocktail, EDTA-free (Thermo Fisher Scientific, 78441). Proteins were then separated using a 4 to 20% Mini-PROTEAN TGX Stain-Free Gel (Bio-Rad, no. 4568094). An anti-ATP5IF1 antibody (Thermo Fisher Scientific, A21355) was used to bind ATP5IF1 protein on the blot membrane, and a secondary anti-mouse antibody was used for chemiluminescent detection of ATP5IF1 expression. Western blot images were analyzed by densitometry using ImageJ v1.53 software, and the density of each ATP5IF1 band was normalized to the density of total protein in each lane to quantify ATP5IF1 expression.

### Lentiviral ATP5IF1 overexpression in AML and HCT116 cells

AML cells were cultured in IMDM (Thermo Fisher Scientific, 31980-030) supplemented with 10% FBS and 1% penicillin/streptomycin, and HCT116 cells were cultured in RPMI 1640 (Thermo Fisher Scientific, 61870-036) supplemented with 10% FBS and 1% penicillin/streptomycin and incubated at 37°C in 5% CO_2_. The Pantropic ViraSafe Universal Lentiviral Expression System (Cell Biolabs Inc., VPK-211-PAN) was used to establish cells overexpressing the human ATP5IF1 and the *Cox8a*MTS:zsGreen1, with *Cox8a* also being derived from human. zsGreen1 is a human codon-optimized variant of *Zoanthus* sp. green fluorescent protein zsGreen. The plasmid used the Ef1-alpha promoter to drive expression of both ATP5IF1 and *Cox8a*MTS:zsGreen1. For antibiotic selection, a puromycin resistance gene was also expressed in the plasmid, driven by the human phosphoglycerate kinase 1 promoter. To facilitate infection, AML cells were cocultured with lentiviral particles at a seeding density of 1 × 10^6^ cells/ml in a RetroNectin dish (Takara Bio, T110A). HCT116 cells were seeded at a density of 0.2 × 10^6^ cells/ml in a six-well plate. Following 72 hours of infection for all cell types, cultures were then subjected to puromycin selection by continuous exposure to puromycin (1 to 2 μg/ml) in the culture medium.

To confirm overexpression of ATP5IF1, mitochondria were first isolated from *Cox8a*MTS:zsGreen1 control cells and ATP5IF1-overexpressing cells. Mitochondria were then lysed using Pierce RIPA Buffer (Thermo Fisher Scientific, 89900) supplemented with 1× Halt Protease and Phosphatase Inhibitor Cocktail, EDTA-free (Thermo Fisher Scientific, 78441). An anti-ATP5IF1 antibody (Thermo Fisher Scientific, A21355) was used to bind ATP5IF1 protein on the blot membrane, and a secondary anti-mouse antibody was used chemiluminescent detection of ATP5IF1 expression. Western blot images were analyzed by densitometry using ImageJ v1.53 software, and the density of each ATP5IF1 band was normalized to the density of total protein in each lane to quantify ATP5IF1 expression. Manipulation of ATP5IF1 was also validated by real-time PCR. To do this, total RNA was extracted using QIAGEN RNeasy Midi kits (QIAGEN, no. 74104). RNA was reverse transcribed using Superscript IV Reverse Transcriptase (Thermo Fisher Scientific, no. 1809105). Real-time PCR on ATP5IF1 was performed using a Quantstudio 3 Real-time PCR system (Thermo Fisher Scientific). Relative quantification of mRNA levels was determined using the comparative threshold cycle (ΔΔCt) method using FAM-labeled TaqMan Gene expression assays specific to ATP5IF1 (Thermo Fisher Scientific, catalog no. 4331182, assay ID no. Hs00603608) run in multiplex with a VIC-labeled 18 s (Thermo Fisher Scientific, catalog no. 4331182, assay ID no. Hs99999901) control primer.

### Determination of ATP hydrolytic activity of complex V

ATPase activity was assessed as described previously with slight modifications (*68*, *71*). Mitochondrial lysates for the assay were prepared via dilution of the final isolated mitochondrial suspensions in CelLytic M (Sigma-Aldrich, C2978) at a protein concentration of 2 mg/ml. Buffer for the assay was ATPase assay buffer, supplemented with lactate dehydrogenase/pyruvate kinase (10 U/ml), phosphoenoyl-pyruvate (5 mM), rotenone (0.005 mM), and NADH (0.2 mM). Assay buffer (200 μl) was loaded into individual wells of a 96-well plate, followed by mitochondrial lysate (2 μg per well). The assay was initiated with the addition of ATP (5 mM). In the assay, NADH oxidation and ATP hydrolysis occur at a 1:1 stoichiometry, and, thus, ATPase activity (represented as a % of control cells) was determined via tracking the degradation in the NADH auto-fluorescence (Ex:Em, 376/450) signal upon ATP addition.

### Western blotting

Isolated mitochondria or intact cells were lysed in Pierce RIPA Buffer (Thermo Fisher Scientific, 89900), supplemented with 1× Halt Protease and Phosphatase Inhibitor Cocktail, EDTA-free (Thermo Fisher Scientific, 78441). Proteins were then separated using a 4 to 20% Mini-PROTEAN TGX Stain-Free Gel (Bio-Rad). Gels were transferred to nitrocellulose membranes, and, then, membranes were blocked in 5% milk in tris-buffered saline for 1 hour. Following blocking, membranes were incubated in primary antibody overnight at 4°C. Primary antibodies were as follows: phosphorylated AMPK (Cell Signaling Technology, no. 2531S), total AMPK (Cell Signaling Technology, no. 2532S), and ATP5IF1 (Thermo Fisher Scientific, no. A21355). Secondary antibodies were horseradish peroxidase–linked anti-rabbit (Cell Signaling Technology, no. 7074) or anti-mouse (Cell Signaling Technology, no. 7076) immunoglobulin G. Thermo Fisher Scientific SuperSignal West Femto Maximum Sensitivity Substrate was used ECL substrate (no. 34095). Bands were detected using chemiluminescence, and quantification was done using ImageJ v1.53 software.

### Determination of intact cell respiration normalized to mitochondrial content

Experimentation on OCI-AML2, MV4-11, and mobilized bone marrow mononuclear cells (BMMCs) was conducted after thawing cells directly from stored, frozen aliquots (−80°C). Each cell type was thawed rapidly in a warm bath (37°C) and then was centrifuged at 300*g*. The supernatant was aspirated, and the cells were resuspended in PBS. Viable cell count was then performed with trypan blue (0.4%) (Thermo Fisher Scientific, 15250-061). From that suspension, 1 × 10^6^ total cells were placed in microcentrifuge tubes and were stored at (−80°C) for proteomics preparation. The remaining cells were centrifuged again at 300*g*. After this, PBS was aspirated, and cells were resuspended in either 0.5 or 1 ml of intact cell respiratory medium [supplemented with 1% penicillin/streptomycin and 10% FBS (pH 7.4)]. Viable cell count was again performed with trypan blue (0.4%) (Thermo Fisher Scientific, 15250-061) for normalization of intact cell respiration. Basal respiration was then quantified, followed by FCCP titration (FC; 0.5 to 5 μM). Antimycin A (Ant A; 0.5 μM) was then added as a negative control to inhibit mitochondrial respiration. At the end of each assay, cell suspensions were removed from the O2k chamber and placed into microcentrifuge tubes. The cell suspension was then centrifuged at 2000*g* for 10 min at 4°C. Assay buffer was aspirated, and the cell pellet was washed with PBS. Cells were then lysed using low-percentage detergent buffer, CelLytic M (Sigma-Aldrich, C2978), followed by a freeze-thaw cycle on dry ice. The protein concentration was then determined using a Pierce BCA protein assay (Sigma-Aldrich, 71285), and intact cell respiration was normalized to milligrams of total intact cell protein.

The frozen 1 × 10^6^ cell aliquots that were stored from each cell type (i.e., OCI-AML2, MV4-11, and mobilized BMMCs) were later used for proteomic analysis. Following proteomics analysis, the ratio of the mitochondrial protein to total protein was used to calculate the mitochondrial enrichment factor (MEF) that provides an estimate of mitochondrial content (*72*). The MEF was then used to normalize intact cell respiration (after normalization to milligrams of total intact cell protein) to the rate of respiration per milligram of total mitochondrial protein.

### Sample prep for label-free proteomics

Cell pellets or isolated mitochondria pellets were lysed in urea lysis buffer [8 M urea in 40 mM tris, 30 mM NaCl, 1 mM CaCl_2_, 1× cOmplete ULTRA mini EDTA-free protease inhibitor tablet (pH 8.0)], as described previously (*23*, *73*). The samples were subjected to two freeze-thaw cycles and sonicated with a probe sonicator in three 5-s bursts (Q Sonica, no. CL-188; amplitude of 30). Samples were centrifuged at 10,000*g* for 10 min at 4°C. Protein concentration was determined by BCA. Equal amounts of protein were reduced with 5 mM dithiothreitol (DTT) at 37°C for 30 min and then alkylated with 15 mM iodoacetamide for 30 min in the dark. Unreacted iodoacetamide was quenched with DTT (15 mM). Reduction and alkylation reaction were carried out at room temperature. Initial digestion was performed with Lys C (1:100 w:w) for 4 hours at 32°C. Following dilution to 1.5 M urea with 40 mM tris (pH 8.0), 30 mM NaCl, and 1 mM CaCl_2_, samples were digested overnight with sequencing grade trypsin (50:1 w/w) at 32°C. Samples were acidified to 0.5% trifluoroacetic acid and then centrifuged at 4000*g* for 10 min at 4°C. Supernatant containing soluble peptides was desalted, and, then, eluate was frozen and subjected to SpeedVac vacuum concentration.

### nLC-MS/MS for label-free proteomics

Final peptides were resuspended in 0.1% formic acid, quantified (Thermo Fisher Scientific, 23275), and diluted to a final concentration of 0.25 μg/μL. Samples were subjected to nano–liquid chromatography–mass spectrometry (nanoLC-MS/MS) analysis using an UltiMate 3000 RSLCnano system (Thermo Fisher Scientific) coupled to a Q Exactive Plus Hybrid Quadrupole-Orbitrap mass spectrometer (Thermo Fisher Scientific) via a nanoelectrospray ionization source. For each injection, 4 μl (1 μg) of sample was first trapped on an Acclaim PepMap 100 20 mm–by–0.075 mm trapping column (Thermo Fisher Scientific, 164535; 5 μl/min at 98/2 v/v water/acetonitrile with 0.1% formic acid). Analytical separation was performed over a 95-min gradient (flow rate of 250 nl/min) of 4 to 25% acetonitrile using a 2-μm EASY-Spray PepMap RSLC C18 75 μm–by–250 mm column (Thermo Fisher Scientific, ES802A) with a column temperature of 45°C. MS1 was performed at 70,000 resolution, with an automatic gain control (AGC) target of 3 × 10^6^ ions and a maximum injection time (IT) of 100 ms. MS2 spectra were collected by data-dependent acquisition of the top 15 most abundant precursor ions with a charge greater than 1 per MS1 scan, with dynamic exclusion enabled for 20 s. Precursor ion isolation window was 1.5 mass/charge ratio (*m*/*z*), and normalized collision energy was 27. MS2 scans were performed at 17,500 resolution, maximum IT of 50 ms, and AGC target of 1 × 10^5^ ions.

### Data analysis for label-free proteomics

Proteome Discoverer 2.2 (PDv2.2) was used for raw data analysis, with default search parameters including oxidation (15.995 Da on methionine) as a variable modification and carbamidomethyl (57.021 Da on cysteine) as a fixed modification. Data were searched against the UniProt *Homo sapiens* reference proteome (Proteome ID: UΡ000005640), as well as the Human MitoCarta 3.0 database (*74*). PSMs were filtered to a 1% false discovery rate (FDR) and grouped to unique peptides while maintaining a 1% FDR at the peptide level. Peptides were grouped to proteins using the rules of strict parsimony and proteins were filtered to 1% FDR. Peptide quantification was done using the MS1 precursor intensity. Imputation was performed via low-abundance resampling. Using only high-confidence master proteins, MEF was determined by comparing mitochondrial protein abundance (i.e., proteins identified to be mitochondrial by cross-reference with the MitoCarta 3.0 database) to total protein abundance.

### Statistical evaluation of label-free proteomics

All proteomics samples were normalized to total protein abundance, and the protein tab in the PDv2.2 results was exported as a tab delimited .txt. file and analyzed. Protein abundance was converted to the log_2_ space. For pairwise comparisons, cell mean, SD, *P* value (*P*; two-tailed Student’s *t* test, assuming equal variance), and adjusted *P* value (Benjamini-Hochberg FDR correction) were calculated. Throughout the paper, differences between groups were assessed by *t* test or one-way analysis of variance (ANOVA), followed by Tukey’s test where appropriate using GraphPad Prism 8 software (version 8.4.2). Other statistical tests used are described in the figure legends. Statistical significance in the figures is indicated as follows: *P < 0.05; **P < 0.01; ***P < 0.001; ****P < 0.0001.

### Metabolite measurement by LC-MS

OCI-AML2 cells were grown under standard culture conditions with indicated drug treatment (1 hour exposure to DMSO or 100 nM venetoclax), and, then, 1 × 10^6^ cells were harvested for metabolite extraction. Before harvesting the cells, the cells were transferred in culture medium to microcentrifuge tubes, cells pelleted, and culture medium aspirated, and, then, 100 μl of cold extraction solvent was added (80:20 mixture of methanol:water with 1 mM of *N*-ethylmaleimide). The tubes were briefly vortexed, incubated on ice for 15 min, and then centrifuged for 30 min at 15,000*g* at 4°C. The supernatant was collected for LC-MS. LC-MS analysis for soluble metabolites was achieved on the quadrupole-orbitrap mass spectrometer Thermo Q Exactive PLUS (Thermo Fisher Scientific). The mass spectrometer was coupled to hydrophilic interaction chromatography via electrospray ionization. The LC separation was performed on a Vanquish Horizon UHPLC system (Thermo Fisher Scientific, Waltham, MA) with an XBridge BEH Amide column (150 mm by 2.1 mm, 2.5-μm particle size, Waters, Milford, MA) using a gradient of solvent A [95%:5% H_2_O:acetonitrile with 20 mM acetic acid, and 40 mM ammonium hydroxide (pH 9.4)] and solvent B [20%:80% H_2_O:acetonitrile with 20 mM acetic acid and 40 mM ammonium hydroxide (pH 9.4)]. The gradient was 0 min, 100% B; 3 min, 100% B; 3.2 min, 90% B; 6.2 min, 90% B; 6.5 min, 80% B; 10.5 min, 80% B; 10.7 min, 70% B; 13.5 min, 70% B; 13.7 min, 45% B; 16 min, 45% B; 16.5 min, 100% B; and 22 min, 100% B. The flow rate was 300 μl/min. The injection volume was 5 μl, and the column temperature was set to 25°C. The autosampler temperature was set to 4°C, and the injection volume was 5 μl. The heated electrospray ionization source of the mass spectrometer was set to a spray voltage of −2.7 kV in negative mode and 3.5 kV in positive mode. The sheath, auxiliary, and sweep gas flow rates were 40, 10, and 2 (arbitrary units), respectively. The capillary temperature was set to 300°C, and the aux gas heater was set to 360°C. The S-lens RF level was 45. The *m*/*z* scan range was set to 72 to 1000 *m*/*z* in either positive or negative ionization mode. The AGC target was set to 3 × 10^6^, and the maximum IT was 200 ms. LC-MS peak files were analyzed and visualized with El-MAVEN (Elucidata) using 5–part-per-million ion extraction window, minimum peak intensity of 1 × 10^4^ ions, and minimum signal to background blank ratio of 2.

### Statistical analysis and software

Statistical analysis was performed using GraphPad Prism 10.1.1. All data are represented as means ± SEM, and analysis was conducted with a significance level set at *P* < 0.05. Details of statistical analysis are included within the figure legends. Figures were created with BioRender.com and GraphPad Prism 10.1.1. Across all figure captions, “*n*” denotes the number of biological replicates.

## References

[1] L. de Beauchamp, E. Himonas, G. V. Helgason, Mitochondrial metabolism as a potential therapeutic target in myeloid leukaemia. Leukemia 36, 1–12 (2022). 34561557 10.1038/s41375-021-01416-w PMC8727299

[2] T. Farge, E. Saland, F. de Toni, N. Aroua, M. Hosseini, R. Perry, C. Bosc, M. Sugita, L. Stuani, M. Fraisse, S. Scotland, C. Larrue, H. Boutzen, V. Feliu, M. L. Nicolau-Travers, S. Cassant-Sourdy, N. Broin, M. David, N. Serhan, A. Sarry, S. Tavitian, T. Kaoma, L. Vallar, J. Iacovoni, L. K. Linares, C. Montersino, R. Castellano, E. Griessinger, Y. Collette, O. Duchamp, Y. Barreira, P. Hirsch, T. Palama, L. Gales, F. Delhommeau, B. H. Garmy-Susini, J. C. Portais, F. Vergez, M. Selak, G. Danet-Desnoyers, M. Carroll, C. Recher, J. E. Sarry, Chemotherapy-resistant human acute myeloid leukemia cells are not enriched for leukemic stem cells but require oxidative metabolism. Cancer Discov. 7, 716–735 (2017). 28416471 10.1158/2159-8290.CD-16-0441 PMC5501738

[3] D. Hou, B. Wang, R. You, X. Wang, J. Liu, W. Zhan, P. Chen, T. Qin, X. Zhang, H. Huang, Stromal cells promote chemoresistance of acute myeloid leukemia cells via activation of the IL-6/STAT3/OXPHOS axis. Ann. Transl. Med. 8, 1346 (2020). 33313091 10.21037/atm-20-3191 PMC7723653

[4] A. U. H. Khan, M. G. Rathore, N. Allende-Vega, D. N. Vo, S. Belkhala, S. Orecchioni, G. Talarico, F. Bertolini, G. Cartron, C. H. Lecellier, M. Villalba, Human leukemic cells performing oxidative phosphorylation (OXPHOS) generate an antioxidant response independently of reactive oxygen species (ROS) production. EBioMedicine 3, 43–53 (2016). 26870816 10.1016/j.ebiom.2015.11.045 PMC4739420

[5] H. J. Park, M. A. Gregory, V. Zaberezhnyy, A. Goodspeed, C. T. Jordan, J. S. Kieft, J. DeGregori, Therapeutic resistance in acute myeloid leukemia cells is mediated by a novel ATM/mTOR pathway regulating oxidative phosphorylation. eLife 11, e79940 (2022). 36259537 10.7554/eLife.79940 PMC9645811

[6] S. Sriskanthadevan, D. V. Jeyaraju, T. E. Chung, S. Prabha, W. Xu, M. Skrtic, B. Jhas, R. Hurren, M. Gronda, X. Wang, Y. Jitkova, M. A. Sukhai, F. H. Lin, N. Maclean, R. Laister, C. A. Goard, P. J. Mullen, S. Xie, L. Z. Penn, I. M. Rogers, J. E. Dick, M. D. Minden, A. D. Schimmer, AML cells have low spare reserve capacity in their respiratory chain that renders them susceptible to oxidative metabolic stress. Blood 125, 2120–2130 (2015). 25631767 10.1182/blood-2014-08-594408 PMC4375109

[7] R. You, D. Hou, B. Wang, J. Liu, X. Wang, Q. Xiao, Z. Pan, D. Li, X. Feng, L. Kang, P. Chen, H. Huang, Bone marrow microenvironment drives AML cell OXPHOS addiction and AMPK inhibition to resist chemotherapy. J. Leukoc. Biol. 112, 299–311 (2022). 34927743 10.1002/JLB.6A0821-409RR PMC9544716

[8] L. Chen, D. Li, X. Guo, C. Cheng, X. Wei, Oridonin synergistically enhances the Pro-apoptotic effect of venetoclax on acute myeloid leukemia cells by inhibiting AKT signaling. Front. Biosci. 28, 195 (2023). 10.31083/j.fbl2809195 37796705

[9] A. Erdem, S. Marin, D. A. Pereira-Martins, M. Geugien, A. Cunningham, M. G. Pruis, I. Weinhauser, A. Gerding, B. M. Bakker, A. T. J. Wierenga, E. M. Rego, G. Huls, M. Cascante, J. J. Schuringa, Inhibition of the succinyl dehydrogenase complex in acute myeloid leukemia leads to a lactate-fuelled respiratory metabolic vulnerability. Nat. Commun. 13, 2013 (2022). 35440568 10.1038/s41467-022-29639-0 PMC9018882

[10] L. Feng, P. Y. Zhang, W. Gao, J. Yu, S. C. Robson, Targeting chemoresistance and mitochondria-dependent metabolic reprogramming in acute myeloid leukemia. Front. Oncol. 13, 1244280 (2023). 37746249 10.3389/fonc.2023.1244280 PMC10513429

[11] F. Liu, H. A. Kalpage, D. Wang, H. Edwards, M. Huttemann, J. Ma, Y. Su, J. Carter, X. Li, L. Polin, J. Kushner, S. H. Dzinic, K. White, G. Wang, J. W. Taub, Y. Ge, Cotargeting of mitochondrial complex I and Bcl-2 shows antileukemic activity against acute myeloid leukemia cells reliant on oxidative phosphorylation. Cancers 12, 2400 (2020). 32847115 10.3390/cancers12092400 PMC7564145

[12] L. Liu, P. K. Patnana, X. Xie, D. Frank, S. C. Nimmagadda, A. Rosemann, M. Liebmann, L. Klotz, B. Opalka, C. Khandanpour, High metabolic dependence on oxidative phosphorylation drives sensitivity to metformin treatment in *MLL/AF9* acute myeloid leukemia. Cancers 14, 486 (2022). 35158754 10.3390/cancers14030486 PMC8833593

[13] S. B. Panina, J. Pei, N. V. Kirienko, Mitochondrial metabolism as a target for acute myeloid leukemia treatment. Cancer Metab. 9, 17 (2021). 33883040 10.1186/s40170-021-00253-w PMC8058979

[14] M. Peng, Y. Huang, L. Zhang, X. Zhao, Y. Hou, Targeting mitochondrial oxidative phosphorylation eradicates acute myeloid leukemic stem cells. Front. Oncol. 12, 899502 (2022). 35574326 10.3389/fonc.2022.899502 PMC9100571

[15] M. Rodriguez-Zabala, R. Ramakrishnan, K. Reinbach, S. Ghosh, L. Oburoglu, A. Falqués-Costa, K. Bellamkonda, M. Ehinger, P. Peña-Martínez, N. Puente-Moncada, H. Lilljebjörn, J. Cammenga, C. J. Pronk, V. Lazarevic, T. Fioretos, A. K. Hagstrom-Andersson, N. B. Woods, M. Järås, Combined GLUT1 and OXPHOS inhibition eliminates acute myeloid leukemia cells by restraining their metabolic plasticity. Blood Adv. 7, 5382–5395 (2023). 37505194 10.1182/bloodadvances.2023009967 PMC10509671

[16] T. A. Yap, N. Daver, M. Mahendra, J. Zhang, C. Kamiya-Matsuoka, F. Meric-Bernstam, H. M. Kantarjian, F. Ravandi, M. E. Collins, M. E. D. Francesco, E. E. Dumbrava, S. Fu, S. Gao, J. P. Gay, S. Gera, J. Han, D. S. Hong, E. J. Jabbour, Z. Ju, D. D. Karp, A. Lodi, J. R. Molina, N. Baran, A. Naing, M. Ohanian, S. Pant, N. Pemmaraju, P. Bose, S. A. Piha-Paul, J. Rodon, C. Salguero, K. Sasaki, A. K. Singh, V. Subbiah, A. M. Tsimberidou, Q. A. Xu, M. Yilmaz, Q. Zhang, Y. Li, C. A. Bristow, M. B. Bhattacharjee, S. Tiziani, T. P. Heffernan, C. P. Vellano, P. Jones, C. J. Heijnen, A. Kavelaars, J. R. Marszalek, M. Konopleva, Complex I inhibitor of oxidative phosphorylation in advanced solid tumors and acute myeloid leukemia: Phase I trials. Nat. Med. 29, 115–126 (2023). 36658425 10.1038/s41591-022-02103-8 PMC11975418

[17] M. E. García Rubiño, E. Carrillo, G. Ruiz Alcalá, A. Domínguez-Martín, J. A. Marchal, H. Boulaiz, Phenformin as an anticancer agent: Challenges and prospects. Int. J. Mol. Sci. 20, 3316 (2019). 31284513 10.3390/ijms20133316 PMC6651400

[18] K. Vasan, N. S. Chandel, Molecular and cellular mechanisms underlying the failure of mitochondrial metabolism drugs in cancer clinical trials. J. Clin. Invest. 134, e176736 (2024). 38299592 10.1172/JCI176736 PMC10836798

[19] X. Zhang, C. V. Dang, Time to hit pause on mitochondria-targeting cancer therapies. Nat. Med. 29, 29–30 (2023). 36658424 10.1038/s41591-022-02129-y PMC11892798

[20] R. Anderson, L. D. Miller, S. Isom, J. W. Chou, K. M. Pladna, N. J. Schramm, L. R. Ellis, D. S. Howard, R. R. Bhave, M. Manuel, S. Dralle, S. Lyerly, B. L. Powell, T. S. Pardee, Phase II trial of cytarabine and mitoxantrone with devimistat in acute myeloid leukemia. Nat. Commun. 13, 1673 (2022). 35354808 10.1038/s41467-022-29039-4 PMC8967916

[21] I. N. Boykov, M. M. Montgomery, J. T. Hagen, R. T. Aruleba, K. L. McLaughlin, H. S. Coalson, M. A. Nelson, A. S. Pereyra, J. M. Ellis, T. N. Zeczycki, N. A. Vohra, S. F. Tan, M. C. Cabot, K. H. Fisher-Wellman, Pan-tissue mitochondrial phenotyping reveals lower OXPHOS expression and function across cancer types. Sci. Rep. 13, 16742 (2023). 37798427 10.1038/s41598-023-43963-5 PMC10556099

[22] L. Chen, M. Zhou, H. Li, D. Liu, P. Liao, Y. Zong, C. Zhang, W. Zou, J. Gao, Mitochondrial heterogeneity in diseases. Signal Transduct. Target. Ther. 8, 311 (2023). 37607925 10.1038/s41392-023-01546-w PMC10444818

[23] M. A. Nelson, K. L. McLaughlin, J. T. Hagen, H. S. Coalson, C. Schmidt, M. Kassai, K. A. Kew, J. M. McClung, P. D. Neufer, P. Brophy, N. A. Vohra, D. Liles, M. C. Cabot, K. H. Fisher-Wellman, Intrinsic OXPHOS limitations underlie cellular bioenergetics in leukemia. eLife 10, e63104 (2021). 34132194 10.7554/eLife.63104 PMC8221809

[24] J. Ngo, C. Osto, F. Villalobos, O. S. Shirihai, Mitochondrial heterogeneity in metabolic diseases. Biology 10, 927 (2021). 34571805 10.3390/biology10090927 PMC8470264

[25] K. H. Fisher-Wellman, J. T. Hagen, M. Kassai, L. P. Kao, M. A. M. Nelson, K. L. McLaughlin, H. S. Coalson, T. E. Fox, S. F. Tan, D. J. Feith, M. Kester, T. P. Loughran Jr., D. F. Claxton, M. C. Cabot, Alterations in sphingolipid composition and mitochondrial bioenergetics represent synergistic therapeutic vulnerabilities linked to multidrug resistance in leukemia. FASEB J. 36, e22094 (2022). 34888943 10.1096/fj.202101194RRR PMC9058980

[26] X. Chen, C. Glytsou, H. Zhou, S. Narang, D. E. Reyna, A. Lopez, T. Sakellaropoulos, Y. Gong, A. Kloetgen, Y. S. Yap, E. Wang, E. Gavathiotis, A. Tsirigos, R. Tibes, I. Aifantis, Targeting mitochondrial structure sensitizes acute myeloid leukemia to venetoclax treatment. Cancer Discov. 9, 890–909 (2019). 31048321 10.1158/2159-8290.CD-19-0117 PMC6606342

[27] R. Dey, C. T. Moraes, Lack of oxidative phosphorylation and low mitochondrial membrane potential decrease susceptibility to apoptosis and do not modulate the protective effect of Bcl-x(L) in osteosarcoma cells. J. Biol. Chem. 275, 7087–7094 (2000). 10702275 10.1074/jbc.275.10.7087

[28] M. Verma, L. Francis, B. N. Lizama, J. Callio, G. Fricklas, K. Z. Q. Wang, B. A. Kaufman, L. D’Aiuto, D. B. Stolz, S. C. Watkins, V. L. Nimgaonkar, A. Soto-Gutierrez, A. Goldstein, C. T. Chu, iPSC-derived neurons from patients with POLG mutations exhibit decreased mitochondrial content and dendrite simplification. Am. J. Pathol. 193, 201–212 (2023). 36414085 10.1016/j.ajpath.2022.11.002 PMC9976192

[29] K. Buchet, C. Godinot, Functional F1-ATPase essential in maintaining growth and membrane potential of human mitochondrial DNA-depleted rho degrees cells. J. Biol. Chem. 273, 22983–22989 (1998). 9722521 10.1074/jbc.273.36.22983

[30] C. Chinopoulos, A. A. Gerencser, M. Mandi, K. Mathe, B. Torocsik, J. Doczi, L. Turiak, G. Kiss, C. Konrad, S. Vajda, V. Vereczki, R. J. Oh, V. Adam-Vizi, Forward operation of adenine nucleotide translocase during F0F1-ATPase reversal: Critical role of matrix substrate-level phosphorylation. FASEB J. 24, 2405–2416 (2010). 20207940 10.1096/fj.09-149898 PMC2887268

[31] F. Di Lisa, R. Menabò, M. Canton, V. Petronilli, The role of mitochondria in the salvage and the injury of the ischemic myocardium. Biochim. Biophys. Acta 1366, 69–78 (1998). 9714744 10.1016/s0005-2728(98)00121-2

[32] Y. Hirabayashi, T. L. Lewis Jr., Y. Du, D. M. Virga, A. M. Decker, G. Coceano, J. Alvelid, M. A. Paul, S. Hamilton, P. Kneis, Y. Takahashi, J. T. Gaublomme, I. Testa, F. Polleux, Most axonal mitochondria in cortical pyramidal neurons lack mitochondrial DNA and consume ATP. bioRxiv 12.579972 [Preprint] (2024). 10.1101/2024.02.12.579972.

[33] D. Okuno, R. Iino, H. Noji, Rotation and structure of FoF1-ATP synthase. J. Biochem. 149, 655–664 (2011). 21524994 10.1093/jb/mvr049

[34] W. W. Chen, K. Birsoy, M. M. Mihaylova, H. Snitkin, I. Stasinski, B. Yucel, E. C. Bayraktar, J. E. Carette, C. B. Clish, T. R. Brummelkamp, D. D. Sabatini, D. M. Sabatini, Inhibition of ATPIF1 ameliorates severe mitochondrial respiratory chain dysfunction in mammalian cells. Cell Rep. 7, 27–34 (2014). 24685140 10.1016/j.celrep.2014.02.046 PMC4040975

[35] E. Gottlieb, S. M. Armour, M. H. Harris, C. B. Thompson, Mitochondrial membrane potential regulates matrix configuration and cytochrome c release during apoptosis. Cell Death Differ. 10, 709–717 (2003). 12761579 10.1038/sj.cdd.4401231

[36] L. D. Zorova, V. A. Popkov, E. Y. Plotnikov, D. N. Silachev, I. B. Pevzner, S. S. Jankauskas, V. A. Babenko, S. D. Zorov, A. V. Balakireva, M. Juhaszova, S. J. Sollott, D. B. Zorov, Mitochondrial membrane potential. Anal. Biochem. 552, 50–59 (2018). 28711444 10.1016/j.ab.2017.07.009 PMC5792320

[37] Y. Xu, H. Ye, Progress in understanding the mechanisms of resistance to BCL-2 inhibitors. Exp. Hematol. Oncol. 11, 31 (2022). 35598030 10.1186/s40164-022-00283-0 PMC9124382

[38] C. D. Di Nardo, B. A. Jonas, V. Pullarkat, M. J. Thirman, J. S. Garcia, A. H. Wei, M. Konopleva, H. Döhner, A. Letai, P. Fenaux, E. Koller, V. Havelange, B. Leber, J. Esteve, J. Wang, V. Pejsa, R. Hájek, K. Porkka, Á. Illés, D. Lavie, R. M. Lemoli, K. Yamamoto, S.-S. Yoon, J.-H. Jang, S.-P. Yeh, M. Turgut, W.-J. Hong, Y. Zhou, J. Potluri, K. W. Pratz, Azacitidine and venetoclax in previously untreated acute myeloid leukemia. N. Engl. J. Med. 383, 617–629 (2020). 32786187 10.1056/NEJMoa2012971

[39] P. Dhakal, M. Bates, M. H. Tomasson, G. Sutamtewagul, A. Dupuy, V. R. Bhatt, Acute myeloid leukemia resistant to venetoclax-based therapy: What does the future hold? Blood Rev. 59, 101036 (2023). 36549969 10.1016/j.blre.2022.101036

[40] B. Nachmias, S. Aumann, A. Haran, A. D. Schimmer, Venetoclax resistance in acute myeloid leukaemia—Clinical and biological insights. Br. J. Haematol. 204, 1146–1158 (2024). 38296617 10.1111/bjh.19314

[41] X. Yue, Q. Chen, J. He, Combination strategies to overcome resistance to the BCL2 inhibitor venetoclax in hematologic malignancies. Cancer Cell Int. 20, 524 (2020). 33292251 10.1186/s12935-020-01614-z PMC7597043

[42] E. D. Lagadinou, A. Sach, K. Callahan, R. M. Rossi, S. J. Neering, M. Minhajuddin, J. M. Ashton, S. Pei, V. Grose, K. M. O’Dwyer, J. L. Liesveld, P. S. Brookes, M. W. Becker, C. T. Jordan, BCL-2 inhibition targets oxidative phosphorylation and selectively eradicates quiescent human leukemia stem cells. Cell Stem Cell 12, 329–341 (2013). 23333149 10.1016/j.stem.2012.12.013 PMC3595363

[43] D. Sharon, S. Cathelin, S. Mirali, J. M. Di Trani, D. J. Yanofsky, K. A. Keon, J. L. Rubinstein, A. D. Schimmer, T. Ketela, S. M. Chan, Inhibition of mitochondrial translation overcomes venetoclax resistance in AML through activation of the integrated stress response. Sci. Transl. Med. 11, eaax2863 (2019). 31666400 10.1126/scitranslmed.aax2863

[44] D. L. Farkas, M. D. Wei, P. Febbroriello, J. H. Carson, L. M. Loew, Simultaneous imaging of cell and mitochondrial membrane potentials. Biophys. J. 56, 1053–1069 (1989). 2611324 10.1016/S0006-3495(89)82754-7 PMC1280610

[45] F. Zanotti, F. Guerrieri, Y. W. Che, R. Scarfò, S. Papa, Proton translocation by the H^+^ -ATPase of mitochondria. Effect of modification by monofunctional reagents of thiol residues in F0 polypeptides. Eur. J. Biochem. 164, 517–523 (1987). 2883005 10.1111/j.1432-1033.1987.tb11157.x

[46] K. Hashiguchi, Q. M. Zhang-Akiyama, Establishment of human cell lines lacking mitochondrial DNA. Methods Mol. Biol. 554, 383–391 (2009). 19513686 10.1007/978-1-59745-521-3_23

[47] S. D. Stuart, A. Schauble, S. Gupta, A. D. Kennedy, B. R. Keppler, P. M. Bingham, Z. Zachar, A strategically designed small molecule attacks alpha-ketoglutarate dehydrogenase in tumor cells through a redox process. Cancer Metab. 2, 4 (2014). 24612826 10.1186/2049-3002-2-4 PMC4108059

[48] Z. Zachar, J. Marecek, C. Maturo, S. Gupta, S. D. Stuart, K. Howell, A. Schauble, J. Lem, A. Piramzadian, S. Karnik, K. Lee, R. Rodriguez, R. Shorr, P. M. Bingham, Non-redox-active lipoate derivates disrupt cancer cell mitochondrial metabolism and are potent anticancer agents in vivo. J. Mol. Med. 89, 1137–1148 (2011). 21769686 10.1007/s00109-011-0785-8

[49] S. B. Panina, N. Baran, F. H. Brasil da Costa, M. Konopleva, N. V. Kirienko, A mechanism for increased sensitivity of acute myeloid leukemia to mitotoxic drugs. Cell Death Dis. 10, 617 (2019). 31409768 10.1038/s41419-019-1851-3 PMC6692368

[50] K. Henkenius, B. H. Greene, C. Barckhausen, R. Hartmann, M. Märken, T. Kaiser, M. Rehberger, S. K. Metzelder, W. J. Parak, A. Neubauer, C. Brendel, E. Mack, Maintenance of cellular respiration indicates drug resistance in acute myeloid leukemia. Leuk. Res. 62, 56–63 (2017). 28985623 10.1016/j.leukres.2017.09.021

[51] K. H. Hurrish, Y. Su, S. Patel, C. L. Ramage, J. Zhao, B. R. Temby, J. L. Carter, H. Edwards, S. A. Buck, S. E. Wiley, M. Huttemann, L. Polin, J. Kushner, S. H. Dzinic, K. White, X. Bao, J. Li, J. Yang, J. Boerner, Z. Hou, G. Al-Atrash, S. N. Konoplev, J. Busquets, S. Tiziani, L. H. Matherly, J. W. Taub, M. Konopleva, Y. Ge, N. Baran, Enhancing anti-AML activity of venetoclax by isoflavone ME-344 through suppression of OXPHOS and/or purine biosynthesis in vitro. Biochem. Pharmacol. 220, 115981 (2024). 38081370 10.1016/j.bcp.2023.115981 PMC11149698

[52] J. V. Bason, M. G. Montgomery, A. G. Leslie, J. E. Walker, Pathway of binding of the intrinsically disordered mitochondrial inhibitor protein to F1-ATPase. Proc. Natl. Acad. Sci. U.S.A. 111, 11305–11310 (2014). 25049402 10.1073/pnas.1411560111 PMC4128166

[53] J. Carroll, I. N. Watt, C. J. Wright, S. Ding, I. M. Fearnley, J. E. Walker, The inhibitor protein IF_1_ from mammalian mitochondria inhibits ATP hydrolysis but not ATP synthesis by the ATP synthase complex. J. Biol. Chem. 300, 105690 (2024). 38280428 10.1016/j.jbc.2024.105690 PMC10906535

[54] R. Kobayashi, H. Ueno, K. I. Okazaki, H. Noji, Molecular mechanism on forcible ejection of ATPase inhibitory factor 1 from mitochondrial ATP synthase. Nat. Commun. 14, 1682 (2023). 37002198 10.1038/s41467-023-37182-9 PMC10066207

[55] H. Döhner, A. H. Wei, F. R. Appelbaum, C. Craddock, C. D. Di Nardo, H. Dombret, B. L. Ebert, P. Fenaux, L. A. Godley, R. P. Hasserjian, R. A. Larson, R. L. Levine, Y. Miyazaki, D. Niederwieser, G. Ossenkoppele, C. Röllig, J. Sierra, E. M. Stein, M. S. Tallman, H.-F. Tien, J. Wang, A. Wierzbowska, B. Löwenberg, Diagnosis and management of AML in adults: 2022 Recommendations from an international expert panel on behalf of the ELN. Blood 140, 1345–1377 (2022). 35797463 10.1182/blood.2022016867

[56] F. Ravandi, S. Pierce, G. Garcia-Manero, T. Kadia, E. Jabbour, G. Borthakur, C. D. DiNardo, N. Daver, N. J. Short, Y. Alvarado, J. Cortes, C. Kim, M. Kelsh, A. Katz, R. Williams, Z. Yang, B. Mehta, H. Kantarjian, Salvage therapy outcomes in a historical cohort of patients with relapsed or refractory acute myeloid leukemia. Clin. Lymphoma Myeloma Leuk. 20, e871–e882 (2020). 32792304 10.1016/j.clml.2020.06.007

[57] P. K. Arnold, L. W. S. Finley, Regulation and function of the mammalian tricarboxylic acid cycle. J. Biol. Chem. 299, 102838 (2023). 36581208 10.1016/j.jbc.2022.102838 PMC9871338

[58] S. Arnold, B. Kadenbach, Cell respiration is controlled by ATP, an allosteric inhibitor of cytochrome-c oxidase. Eur. J. Biochem. 249, 350–354 (1997). 9363790 10.1111/j.1432-1033.1997.t01-1-00350.x

[59] P. Sun, T. Bai, T. Ma, J. Ding, Molecular mechanism of the dual regulatory roles of ATP on the αγ heterodimer of human NAD-dependent isocitrate dehydrogenase. Sci. Rep. 10, 6225 (2020). 32277159 10.1038/s41598-020-63425-6 PMC7148312

[60] A. D. Zimmer, G. Walbrecq, I. Kozar, I. Behrmann, C. Haan, Phosphorylation of the pyruvate dehydrogenase complex precedes HIF-1-mediated effects and pyruvate dehydrogenase kinase 1 upregulation during the first hours of hypoxic treatment in hepatocellular carcinoma cells. Hypoxia 4, 135–145 (2016). 27800515 10.2147/HP.S99044 PMC5085306

[61] E. N. Maldonado, J. J. Lemasters, ATP/ADP ratio, the missed connection between mitochondria and the Warburg effect. Mitochondrion 19 ( Pt. A), 78–84 (2014). 25229666 10.1016/j.mito.2014.09.002 PMC4254332

[62] J. B. McMillin, D. F. Pauly, Control of mitochondrial respiration in muscle. Mol. Cell. Biochem. 81, 121–129 (1988). 3050450 10.1007/BF00219314

[63] B. Wiench, T. Eichhorn, M. Paulsen, T. Efferth, Shikonin directly targets mitochondria and causes mitochondrial dysfunction in cancer cells. Evid. Based Complement. Alternat. Med. 2012, 726025 (2012). 23118796 10.1155/2012/726025 PMC3478753

[64] E. M. Stein, C. D. Di Nardo, A. T. Fathi, A. S. Mims, K. W. Pratz, M. R. Savona, A. S. Stein, R. M. Stone, E. S. Winer, C. S. Seet, H. Döhner, D. A. Pollyea, J. K. Mc Closkey, O. Odenike, B. Löwenberg, G. J. Ossenkoppele, P. A. Patel, M. Roshal, M. G. Frattini, F. Lersch, A. Franovic, S. Nabhan, B. Fan, S. Choe, H. Wang, B. Wu, L. Hua, C. Almon, M. Cooper, H. M. Kantarjian, M. S. Tallman, Ivosidenib or enasidenib combined with intensive chemotherapy in patients with newly diagnosed AML: A phase 1 study. Blood 137, 1792–1803 (2021). 33024987 10.1182/blood.2020007233 PMC8020270

[65] B. J. Reisman, H. Guo, H. E. Ramsey, M. T. Wright, B. I. Reinfeld, P. B. Ferrell, G. A. Sulikowski, W. K. Rathmell, M. R. Savona, L. Plate, J. L. Rubinstein, B. O. Bachmann, Apoptolidin family glycomacrolides target leukemia through inhibition of ATP synthase. Nat. Chem. Biol. 18, 360–367 (2022). 34857958 10.1038/s41589-021-00900-9 PMC8967781

[66] H. S. Brunetta, A. S. Jung, F. Valdivieso-Rivera, S. C. de Campos Zani, J. Guerra, V. O. Furino, A. Francisco, M. Bercot, P. M. Moraes-Vieira, S. Keipert, M. Jastroch, L. O. Martinez, C. H. Sponton, R. F. Castilho, M. A. Mori, A. Bartelt, IF1 is a cold-regulated switch of ATP synthase hydrolytic activity to support thermogenesis in brown fat. EMBO J. 43, 4870–4891 (2024). 39284909 10.1038/s44318-024-00215-0 PMC11535227

[67] S. Zheng, W. Wang, J. Aldahdooh, A. Malyutina, T. Shadbahr, Z. Tanoli, A. Pessia, J. Tang, SynergyFinder Plus: Toward better interpretation and annotation of drug combination screening datasets. Genomics Proteomics Bioinformatics 20, 587–596 (2022). 35085776 10.1016/j.gpb.2022.01.004 PMC9801064

[68] K. H. Fisher-Wellman, M. T. Davidson, T. M. Narowski, C. T. Lin, T. R. Koves, D. M. Muoio, Mitochondrial diagnostics: A multiplexed assay platform for comprehensive assessment of mitochondrial energy fluxes. Cell Rep. 24, 3593–3606.e10 (2018). 30257218 10.1016/j.celrep.2018.08.091 PMC6237617

[69] D. R. Stirling, M. J. Swain-Bowden, A. M. Lucas, A. E. Carpenter, B. A. Cimini, A. Goodman, CellProfiler 4: Improvements in speed, utility and usability. BMC Bioinformatics 22, 433 (2021). 34507520 10.1186/s12859-021-04344-9 PMC8431850

[70] C. A. Schneider, W. S. Rasband, K. W. Eliceiri, NIH Image to ImageJ: 25 years of image analysis. Nat. Methods 9, 671–675 (2012). 22930834 10.1038/nmeth.2089 PMC5554542

[71] A. Barrientos, F. Fontanesi, F. Diaz, Evaluation of the mitochondrial respiratory chain and oxidative phosphorylation system using polarography and spectrophotometric enzyme assays. Curr. Protoc. Hum. Genet., 10.1002/0471142905.hg1903s63, (2009). 10.1002/0471142905.hg1903s63 PMC2771113 19806590

[72] K. L. McLaughlin, J. T. Hagen, H. S. Coalson, M. A. M. Nelson, K. A. Kew, A. R. Wooten, K. H. Fisher-Wellman, Novel approach to quantify mitochondrial content and intrinsic bioenergetic efficiency across organs. Sci. Rep. 10, 17599 (2020). 33077793 10.1038/s41598-020-74718-1 PMC7572412

[73] K. L. McLaughlin, K. A. Kew, J. M. McClung, K. H. Fisher-Wellman, Subcellular proteomics combined with bioenergetic phenotyping reveals protein biomarkers of respiratory insufficiency in the setting of proofreading-deficient mitochondrial polymerase. Sci. Rep. 10, 3603 (2020). 32107436 10.1038/s41598-020-60536-y PMC7046634

[74] S. Rath, R. Sharma, R. Gupta, T. Ast, C. Chan, T. J. Durham, R. P. Goodman, Z. Grabarek, M. E. Haas, W. H. W. Hung, P. R. Joshi, A. A. Jourdain, S. H. Kim, A. V. Kotrys, S. S. Lam, J. G. McCoy, J. D. Meisel, M. Miranda, A. Panda, A. Patgiri, R. Rogers, S. Sadre, H. Shah, O. S. Skinner, T.-L. To, M. A. Walker, H. Wang, P. S. Ward, J. Wengrod, C. C. Yuan, S. E. Calvo, V. K. Mootha, MitoCarta3.0: An updated mitochondrial proteome now with sub-organelle localization and pathway annotations. Nucleic Acids Res. 49, D1541–D1547 (2021). 33174596 10.1093/nar/gkaa1011 PMC7778944

[75] E. W. Deutsch, A. Csordas, Z. Sun, A. Jarnuczak, Y. Perez-Riverol, T. Ternent, D. S. Campbell, M. Bernal-Llinares, S. Okuda, S. Kawano, R. L. Moritz, J. J. Carver, M. Wang, Y. Ishihama, N. Bandeira, H. Hermjakob, J. A. Vizcaíno, The ProteomeXchange consortium in 2017: Supporting the cultural change in proteomics public data deposition. Nucleic Acids Res. 45, D1100–D1106 (2017). 27924013 10.1093/nar/gkw936 PMC5210636

[76] S. Okuda, Y. Watanabe, Y. Moriya, S. Kawano, T. Yamamoto, M. Matsumoto, T. Takami, D. Kobayashi, N. Araki, A. C. Yoshizawa, T. Tabata, N. Sugiyama, S. Goto, Y. Ishihama, jPOSTrepo: An international standard data repository for proteomes. Nucleic Acids Res. 45, D1107–D1111 (2017). 27899654 10.1093/nar/gkw1080 PMC5210561

