# Supplementary material for: Acute myeloid leukemia mitochondria hydrolyze ATP to support oxidative metabolism and resist chemotherapy
Source: Sci Adv. 2025 Apr 9;11(15):eadu5511. doi: 10.1126/sciadv.adu5511 (PMC11980858; doi:10.1126/sciadv.adu5511)
Supplement: Supplementary file 1 — Figs. S1 to S6 Legends for tables S1 and S2 [file sciadv.adu5511_sm.pdf]

Science Advances
AAAS

Supplementary Materials for

Acute myeloid leukemia mitochondria hydrolyze ATP to support oxidative metabolism and resist chemotherapy

James T. Hagen *et al.*

Corresponding author: Kelsey H. Fisher-Wellman, kfisherw@wakehealth.edu

Sci. Adv. **11**, eadu5511 (2025)
DOI: 10.1126/sciadv.adu5511

The PDF file includes:

Figs. S1 to S6
Legends for tables S1 and S2

Other Supplementary Material for this manuscript includes the following:

Tables S1 and S2

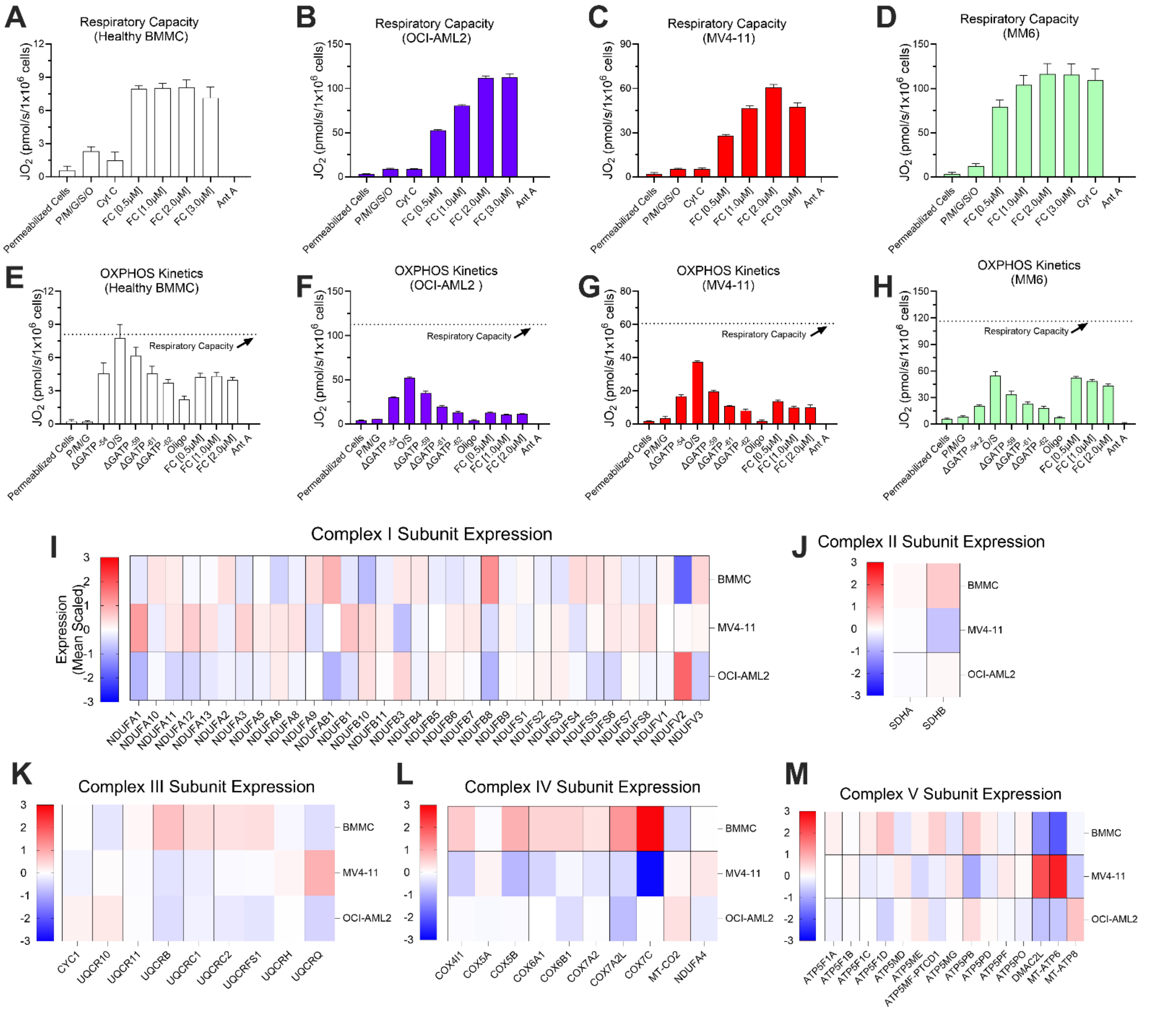

Supplementary Figure 1. Respiratory capacity and OxPhos kinetics profiling of AML and healthy hematopoietic cells. (Related to Figure 1)

All experiments were performed using whole intact or digitonin-permeabilized cells. Respiratory capacity was measured using (A) Healthy BMMC (*N* = 3 replicates), (B) AML2$_{WT}$ (*N* = 3 replicates), (C) MV4-11 (*N* = 3 replicates), (D) MM6 cells. OXPHOS respiratory kinetics were measured using (E) Healthy BMMC (*N* = 3 replicates), (F) OCI-AML2 (*N* = 3 replicates), (G) MV4-11 (*N* = 3 replicates), (H) MM6 cells (*N* = 3 replicates). (I) Comparison of Complex I subunit expression in Mobilized BMMC and AML cells (*N* = 3 replicates). (J) Comparison of Complex II subunit expression in Mobilized BMMC and AML cells (*N* = 3 replicates). (K) Comparison of Complex III subunit expression in Mobilized BMMC and AML cells (*N* = 3 replicates). (L) Comparison of Complex IV subunit expression in Mobilized BMMC and AML cells (*N* = 3 replicates). (M) Comparison of Complex V subunit expression in Mobilized BMMC and AML cells (*N* = 3 replicates).

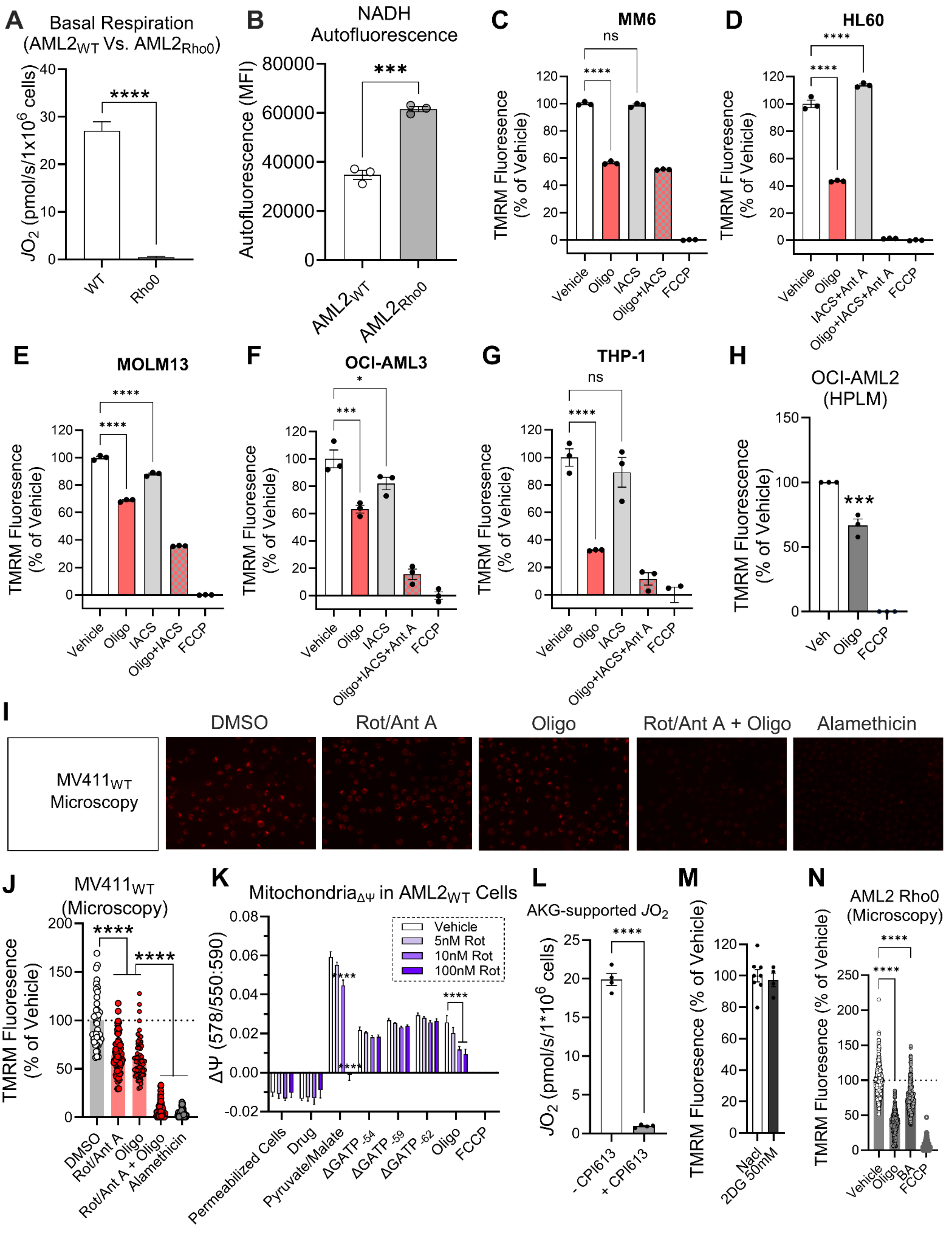

Supplementary Figure 2. Extended validation of ρ^0 OCI-AML2 cells and F_1-ATPase activity in AML cells. (Related to Figure 2)

All experiments were performed using whole intact cells or isolated mitochondria. (A) Comparison of basal respiration of $AML2_{Rho0}$ or $AML2_{WT}$ cells (N = 4 replicates). (B) Comparison of NADH autofluorescence of $AML2_{Rho0}$ or $AML2_{WT}$ cells (N = 3 replicates). (C-G) Flow cytometric analysis of intact cell $\Delta\Psi_m$ in various AML cells assayed following exposure to the indicated inhibitors (N = 3 replicates). (H) Flow cytometric analysis of intact cell $\Delta\Psi_m$ in $AML2_{WT}$ cells assayed in human plasma-like media (HPLM) (N = 3 replicates). (I) Fluorescent microscopy representative images of intact cell $\Delta\Psi_m$ in $MV411_{WT}$ cells. (J) Fluorescent microscopy analysis of intact cell $\Delta\Psi_m$ in $MV411_{WT}$ cells (N = 39-154 cells). (K) Effect of rotenone on permeabilized $AML2_{WT}$ cell $\Delta\Psi_m$ (N = 4 replicates). (L) Permeabilized cell respiration supported by alpha-ketoglutarate (10mM) in the absence and presence of CPI-613 (200μM) in MOLM13 cells (N = 4 replicates). (M) Flow cytometric analysis of intact cell $\Delta\Psi_m$ in MOLM13 cells following short-term (15 minutes) exposure to NaCl (50mM) or 2-DG (50mM) (N = 3-8 replicates). (N) Fluorescent microscopy analysis of intact cell $\Delta\Psi_m$ in $AML2_{Rho0}$ cells in the presence of DMSO, bongkrekic acid (25μM), oligomycin (10μM), or FCCP (10μM) (N = 251-435 cells). Data are presented as mean ±SEM and analyzed by two-way ANOVA (K), one-way ANOVA (C-H,J,N), or unpaired t-test (A,B,L,M). $^{*}p<0.05$, $^{**}p<0.01$, $^{***}p<0.001$, $^{****}p<0.0001$.

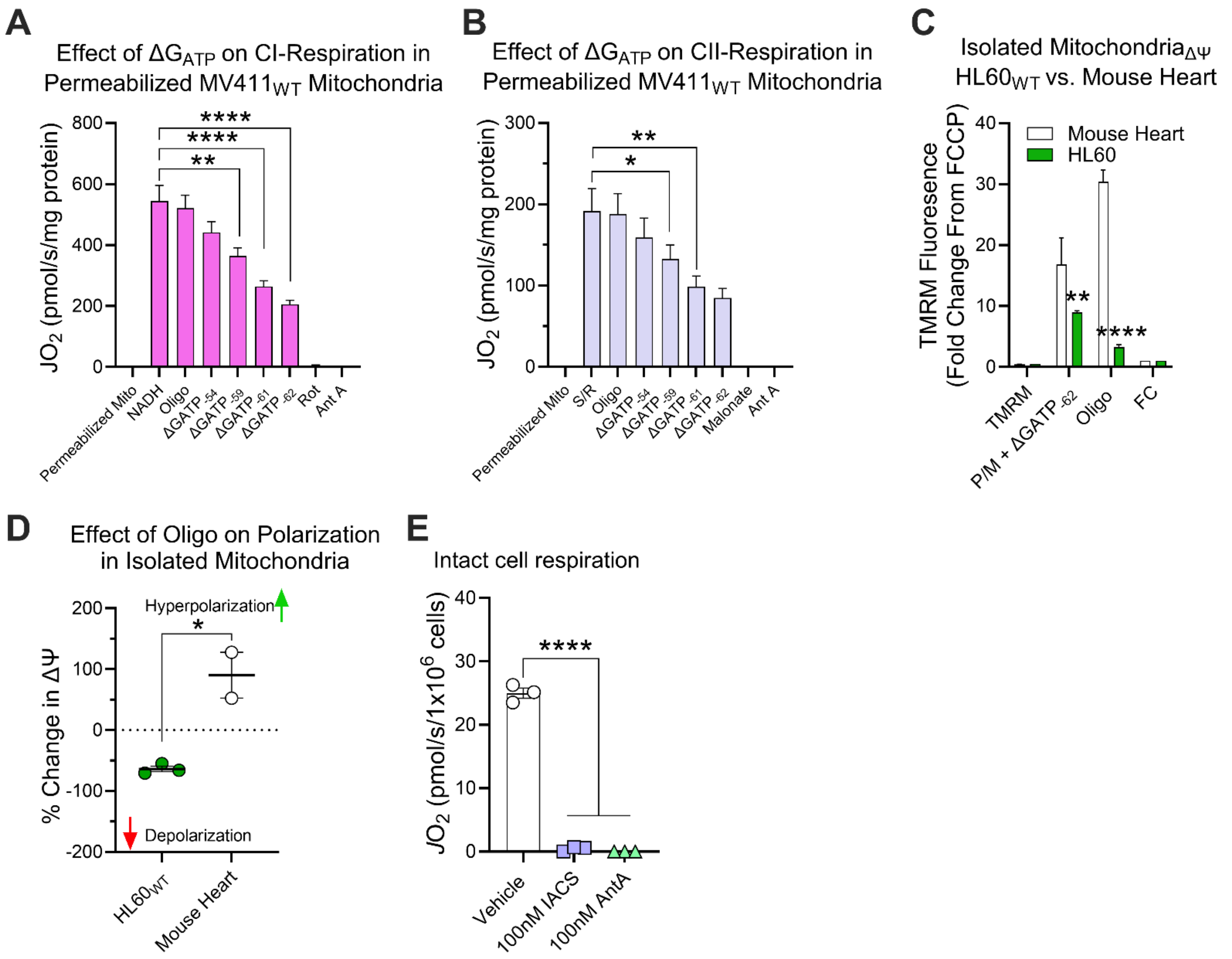

Supplementary Figure 3. Extended validation of the potential mechanism of oligomycin-induced mitochondrial depolarization and cell apoptosis. (Related to Figure 3)

All experiments were performed using whole intact cells, digitonin-permeabilized cells, or isolated mitochondria. (A) Effect of ΔG_{ATP} on NADH (Complex I) supported respiration in alamethicin permeabilized isolated $MV411_{WT}$ mitochondria (*N* = 4 replicates). (B) Effect of ΔG_{ATP} on succinate (Complex II) supported respiration in alamethicin permeabilized isolated $MV411_{WT}$ mitochondria (*N* = 4 replicates). (C) Flow cytometric analysis of the $\Delta\Psi_m$ of isolated mitochondria derived from mouse heart or HL60 cells (*N* = 2-3 replicates). (D) Comparison of oligomycin induced polarization of isolated mitochondria derived from mouse heart or HL60 cells (*N* = 2-3 replicates). (E) Effect of IACS or Ant A on $AML2_{WT}$ basal respiration (*N* = 3 replicates). Data are presented as mean ±SEM and analyzed by one-way ANOVA (A,B,E), or unpaired t-test (C,D). *p<0.05, **p<0.01, ***p<0.001, ****p<0.0001.

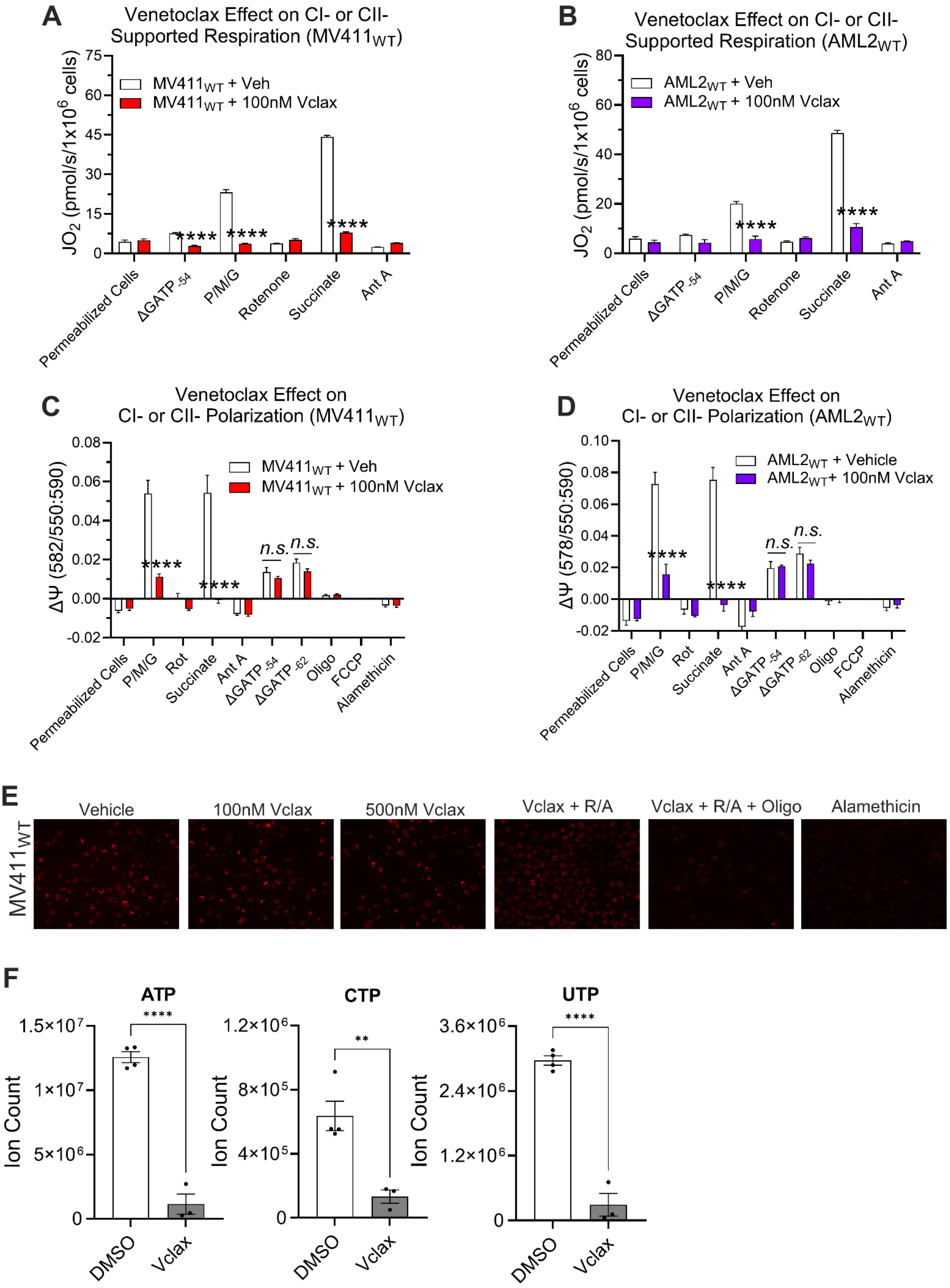
A
Venetoclax Effect on CI- or CII- Supported Respiration (MV411WT)
MV411WT + Veh
MV411WT + 100nM Vclax
JO2 (pmol/s/1x10^6 cells)
Permeabilized Cells
ΔGATP -54
P/M/G
Rotenone
Succinate
Ant A
B
Venetoclax Effect on CI- or CII- Supported Respiration (AML2WT)
AML2WT + Veh
AML2WT + 100nM Vclax
C
Venetoclax Effect on CI- or CII- Polarization (MV411WT)
ΔΨ (582/550:590)
n.s.
Rot
ΔGATP -62
Oligo
FCCP
Alamethicin
D
Venetoclax Effect on CI- or CII- Polarization (AML2WT)
AML2WT + Vehicle
AML2WT+ 100nM Vclax
ΔΨ (578/550:590)
E
Vehicle
100nM Vclax
500nM Vclax
Vclax + R/A
Vclax + R/A + Oligo
Alamethicin
MV411WT
F
ATP
CTP
UTP
Ion Count
DMSO
Vclax

Supplementary Figure 4. Complete and representative data for the experiments involving venetoclax exposure. (Related to Figure 4)

All experiments were performed using whole intact cells or digitonin-permeabilized cells. (A) Effect of 1 hour exposure to 100nM venetoclax on respiration supported by Complex I substrates (P/M/G) or Complex II substrates (S/R) in $MV411_{WT}$ cells (N = 4 replicates). (B) Effect of 1 hour exposure to 100nM venetoclax on respiration supported by Complex I substrates (P/M/G) or Complex II substrates (S/R) in $AML2_{WT}$ cells (N = 4 replicates). (C) Effect of 1 hour exposure to 100nM venetoclax on polarization stimulated by Complex I substrates (P/M/G) or Complex II substrates (S/R) in $MV411_{WT}$ cells (N = 6 replicates). (D) Effect of 1 hour exposure to 100nM venetoclax on polarization stimulated by Complex I substrates (P/M/G) or Complex II substrates (S/R) in $AML2_{WT}$ cells (N = 4 replicates). (E) Fluorescent microscopy images of $\Delta\Psi_m$ in $MV411_{WT}$ cells exposed to venetoclax, venetoclax in combination with rotenone and antimycin A, or venetoclax in combination with rotenone, antimycin A, and oligomycin. (F) Intracellular ATP, CTP, and UTP in OCI-AML2 cells exposed to DMSO or venetoclax (100nM) for 1-hour (N = 3 replicates). Data are presented as mean ±SEM and analyzed by two-way ANOVA (A,B,C,D), unpaired t-test (F). *$p<0.05$, **$p<0.01$, ***$p<0.001$, ****$p<0.0001$.

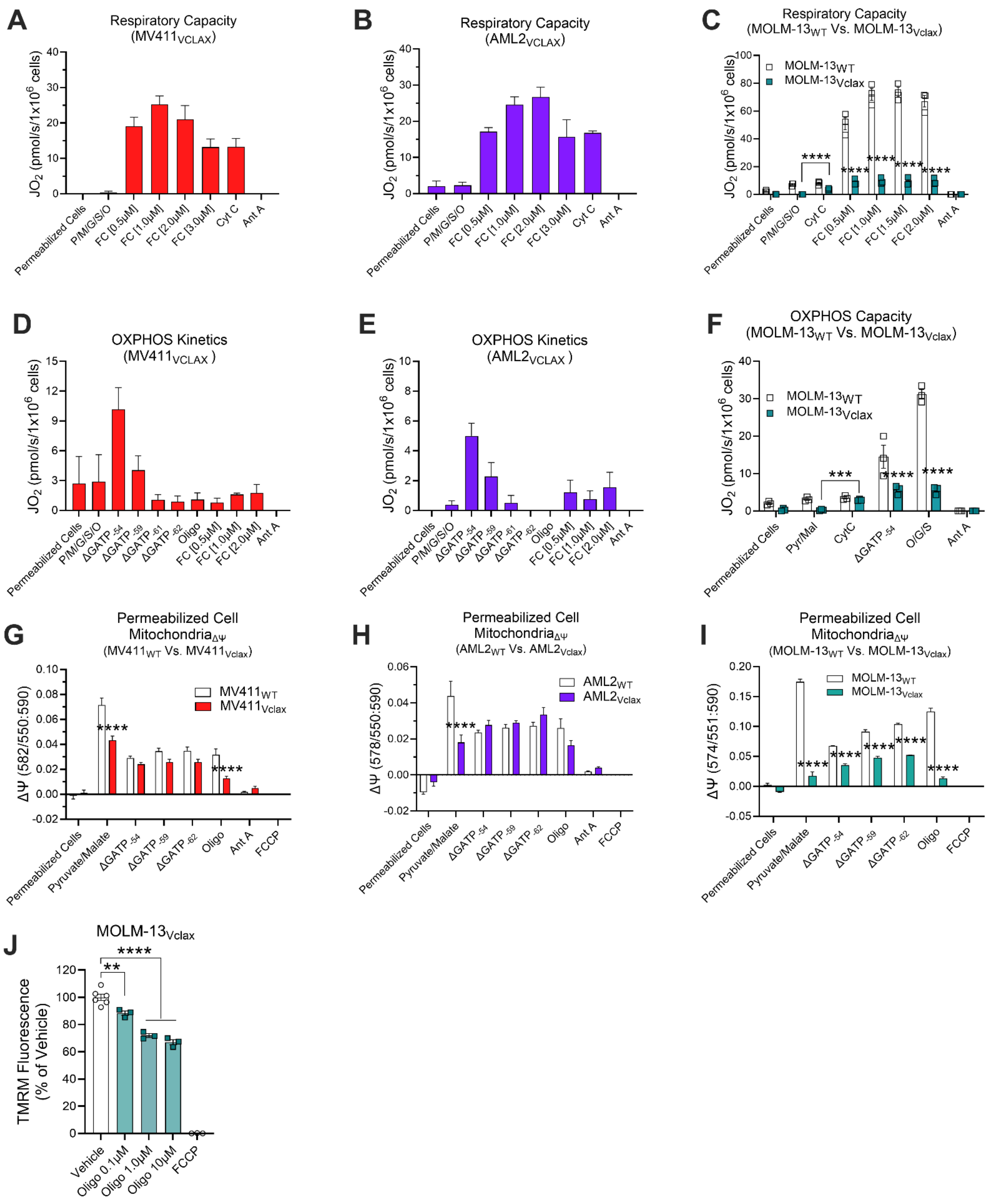

Supplementary Figure 5. Complete and representative data for the experiments involving venetoclax resistance. (Related to Figure 5)

All experiments were performed using digitonin-permeabilized cells. (A) Assessment of respiratory capacity in permeabilized MV411$_{Vclax}$ cells (*N* = 3 replicates). (B) Assessment of respiratory capacity in permeabilized AML2$_{Vclax}$ cells (*N* = 3 replicates). (C) Comparison of respiratory capacity in permeabilized MOLM-13$_{WT}$ cells or MOLM-13$_{Vclax}$ cells (*N* = 3 replicates). (D) Assessment of OxPhos respiratory kinetics in permeabilized MV411$_{Vclax}$ cells (*N* = 3 replicates). (E) Assessment of OxPhos respiratory kinetics in permeabilized AML2$_{Vclax}$ cells (*N* = 3 replicates). (F) Comparison of OxPhos capacity in permeabilized MOLM-13$_{WT}$ cells or MOLM-13$_{Vclax}$ cells (*N* = 3 replicates). (G) Comparison of the $\Delta\Psi_m$ in permeabilized MV411$_{WT}$ cells and MV411$_{Vclax}$ cells (*N* = 5-6 replicates). (H) Comparison of the $\Delta\Psi_m$ in permeabilized AML2$_{WT}$ cells and AML2$_{Vclax}$ cells (*N* = 5 replicates). (I) Comparison of the $\Delta\Psi_m$ in permeabilized MOLM-13$_{WT}$ cells and MOLM-13$_{Vclax}$ cells (*N* = 3 replicates). (J) Flow cytometric analysis of $\Delta\Psi_m$ in intact MOLM-13$_{Vclax}$ cells in the presence of oligomycin (*N* = 3-6 replicates). Data are presented as mean ±SEM and analyzed by two-way ANOVA (C,F,G,H,I) or one-way ANOVA (J). *$p<0.05$, **$p<0.01$, ***$p<0.001$, ****$p<0.0001$.

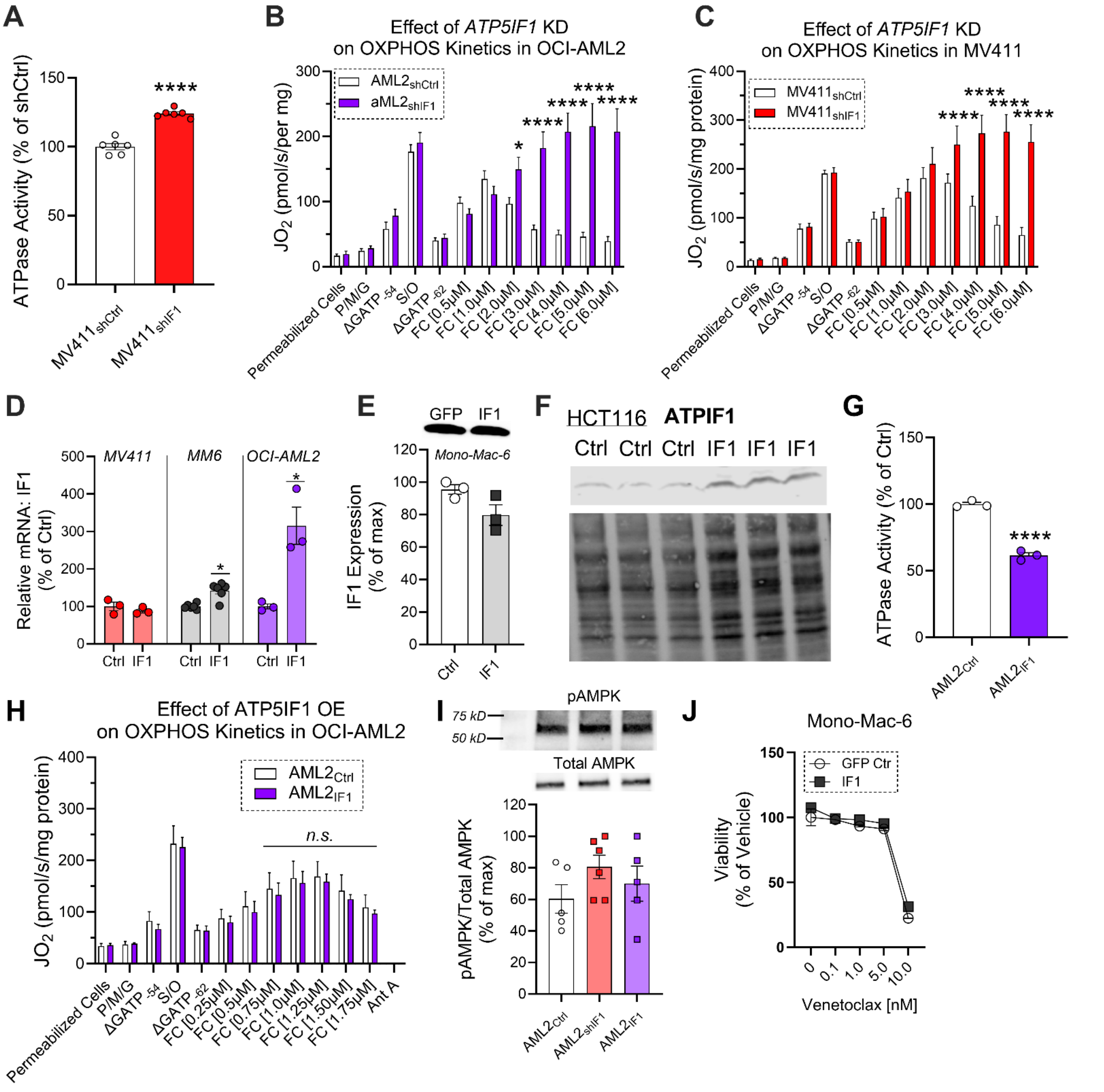

Supplementary Figure 6. Extended characterization of ATP5IF1 loss-of-function and gain-of-function models. (Related to Figures 6 and 7)

(A) Comparison of ATPase activity in isolated mitochondria lysates derived from $MV411_{shCtrl}$ and $MV411_{shIF1}$ cells (N = 6 replicates). (B) Comparison of OxPhos respiratory kinetics in permeabilized $AML2_{shCtrl}$ cells or $AML2_{shIF1}$ cells (N = 8-14 replicates). (C) Comparison of OxPhos respiratory kinetics in permeabilized $MV411_{shCtrl}$ cells or $MV411_{shIF1}$ cells (N = 9-10 replicates). (D) Relative ATP5IF1 mRNA in vector control cells compared to cells infected with lentiviral vectors driving expression of ATP5IF1 (N = 3-6 replicates). (E) Protein abundance of ATP5IF1 in $MM6_{Ctrl}$ vs $MM6_{IF1}$ (N = 3 replicates). (F) Western blot image of ATP5IF1 expression in mitochondria isolated from $HCT116_{Ctrl}$ and HCT_{IF1} cells (N = 3 replicates). (G) Comparison of ATPase activity in isolated mitochondria lysates derived from $AML2_{Ctrl}$ and $AML2_{IF1}$ cells (N = 6 replicates) (H) Comparison of OxPhos respiratory kinetics in permeabilized $AML2_{Ctrl}$ cells or $AML2_{IF1}$ cells (N = 4 replicates). (I) Expression

of phosphorylated AMPK relative to total AMPK in $AML2_{Ctrl}$, $AML2_{shIF1}$, and $AML2_{IF1}$. Expression relative to total AMPK (*N* = 5-6 replicates). (J) Cell viability in response to 48hr exposure to venetoclax in $MM6_{Ctrl}$ vs $MM6_{IF1}$ (*N* = 3 replicates). Data are presented as mean ±SEM and analyzed by two-way ANOVA (B,C,H), one-way ANOVA (I), or unpaired t-test (A,D,E,G,J). *$p<0.05$, **$p<0.01$, ***$p<0.001$, ****$p<0.0001$.

Supplemental Table 1. Individual data points for all figures.

Supplemental Table 2. Proteomics Data. (**A**) Exported results from Proteome Discoverer 2.2 using the whole human proteome database. (**B**) Analyzed results, inclusive of Mitochondrial Enrichment Factor (MEF) calculation. (**C**) Exported results from Proteome Discoverer 2.2 using the human MitoCarta 3.0 database. (**D**) Analyzed results for MitoCarta 3.0 positive proteins.
